# Ethnic minority disparities in progression and mortality of pre-dialysis chronic kidney disease: a systematic scoping review

**DOI:** 10.1186/s12882-020-01852-3

**Published:** 2020-06-09

**Authors:** Hilda O. Hounkpatin, Simon D. S. Fraser, Rory Honney, Gavin Dreyer, Alison Brettle, Paul J. Roderick

**Affiliations:** 1 School of Primary Care, Population Sciences and Medical Education, Faculty of Medicine, South Academic Block, University of Southampton, Southampton General Hospital, Tremona Road, Room AC18 Level C, Southampton, SO16 6YD UK; 2 Department of Nephrology, Barts Health NHS Trust, London, UK; 3 School of Nursing, Midwifery, Social Work and Social Sciences, University of Salford, Rm 1.47, Mary Seacole Building, Frederick Road, Salford, M6 6PU UK

**Keywords:** Chronic kidney disease, Epidemiology, End stage renal disease, Ethnicity, Pre-dialysis

## Abstract

**Background:**

There are a growing number of studies on ethnic differences in progression and mortality for pre-dialysis chronic kidney disease (CKD), but this literature has yet to be synthesised, particularly for studies on mortality.

**Methods:**

This scoping review synthesized existing literature on ethnic differences in progression and mortality for adults with pre-dialysis CKD, explored factors contributing to these differences, and identified gaps in the literature. A comprehensive search strategy using search terms for ethnicity and CKD was taken to identify potentially relevant studies. Nine databases were searched from 1992 to June 2017, with an updated search in February 2020.

**Results:**

8059 articles were identified and screened. Fifty-five studies (2 systematic review, 7 non-systematic reviews, and 46 individual studies) were included in this review. Most were US studies and compared African-American/Afro-Caribbean and Caucasian populations, and fewer studies assessed outcomes for Hispanics and Asians. Most studies reported higher risk of CKD progression in Afro-Caribbean/African-Americans, Hispanics, and Asians, lower risk of mortality for Asians, and mixed findings on risk of mortality for Afro-Caribbean/African-Americans and Hispanics, compared to Caucasians. Biological factors such as hypertension, diabetes, and cardiovascular disease contributed to increased risk of progression for ethnic minorities but did not increase risk of mortality in these groups.

**Conclusions:**

Higher rates of renal replacement therapy among ethnic minorities may be partly due to increased risk of progression and reduced mortality in these groups. The review identifies gaps in the literature and highlights a need for a more structured approach by researchers that would allow higher confidence in single studies and better harmonization of data across studies to advance our understanding of CKD progression and mortality.

## Background

Chronic kidney disease (CKD) is common and is associated with increased morbidity and mortality [1–3]. Risk factors for progression include proteinuria, comorbid conditions such as diabetes and cardiovascular disease, as well as non-modifiable characteristics such as ethnicity [4, 5]. In the UK, a higher proportion of people from ethnic minority groups than Caucasians begin renal replacement therapy (RRT) [6]. In the United States (US), the rate of RRT initiation for end-stage kidney disease (ESKD) is also disproportionately higher for ethnic minority groups (such as African-American, Hispanic and Native Americans) compared to Caucasians, despite similar prevalence for early stages of CKD [7]. Higher RRT prevalence in ethnic minority groups has been attributed to faster progression of CKD and better CKD survival [7, 8]. Understanding which ethnic groups have worse outcomes and which factors influence adverse outcomes can help clinicians and policy makers target health care efforts and resources and improve outcomes for individuals, as well as inform policies to reduce health inequities.

To our knowledge, no study has systematically scoped studies exploring the range of risk factors for ethnic differences in CKD progression and mortality. A systematic review published in 2010 [9] that investigated ethnic differences in CKD progression, and had similar inclusion criteria to the current study, identified 5 relevant studies and concluded little evidence for ethnic differences in CKD progression and a lack of appropriately designed studies to assess ethnic differences in CKD progression. However, there have since been further studies in the field. Furthermore, no studies have systematically synthesised evidence for ethnic differences in mortality for people with pre-dialysis CKD, which would further contribute to understanding of ethnic differences in CKD progression – a competing risk. A scoping review rapidly examines the extent, range, and nature of existing knowledge in a diverse body of literature, summarises research findings and identifies research gaps [10]. In contrast to systematic reviews, scoping reviews identify and synthesise the breadth of knowledge in a given area without in depth assessment of study quality. The aim of this scoping review was to identify and present findings from studies addressing ethnic differences in pre-dialysis CKD progression and mortality, the key factors that underpin these ethnic differences, and identify areas where further research is needed. Understanding the patterns and potential mechanisms in ethnic minorities in high-income countries may contribute to CKD prevention and care in countries where they are the main ethnic groups. We conducted a scoping review to synthesize the existing literature comparing CKD progression and mortality for ethnic minority and non-ethnic minority adults with pre-dialysis CKD.

## Methods

Our scoping review was conducted in line with the five-stage framework outlined by Arskey and O’Malley (2005) [10]. This framework includes formulating the research question, identifying relevant studies, study selection, charting the data, and collating, summarizing and reporting results. We acknowledge that classification of ethnicity can be complex and challenging. In this study, ethnic minority was defined as belonging to a particular group of people with common social, physical, national, linguistic, cultural, ancestral backgrounds and other such attributes living in a country where they differ from the majority [11]. Ethnic minority groups include African-Americans, Hispanic, Asian (East Asia, Southeast Asia, Indian subcontinent), Native Hawaiian or Pacific Islanders, and American Indian or Alaska Native in US studies, and Afro-Caribbean, South Asian (comprising Indians, Pakistanis, Bangladeshis), East Asians [e.g.: Chinese], and other Asian countries, and First Nation populations in UK and other countries [e.g.: Aboriginal Australians]. Pre-dialysis CKD was defined by glomerular filtration rate (GFR) or a combination of urinary albumin to creatinine ratio and GFR, and not requiring RRT.

### Information sources and search strategies

Search terms for ethnic groups included ‘ethnic groups, race, minority, Asian, Caucasian, Hispanic, Continental population groups, and African’ [11]. Search terms for CKD included ‘kidney diseases, renal insufficiency, glomerular filtration rate’. Searches were expanded using truncation symbols and search terms were combined using Boolean operators. The electronic databases Medline OVID, Embase, CINAHL, PsycINFO, Web of Science, Scopus, Social Care Online, Applied Social Sciences Index and Abstracts (ASSIA), and Cochrane Database of Promoting Health Effectiveness Reviews were searched from 1992 to July 2017. An updated search was conducted in February 2020 to identify more recent eligible studies. A time frame of 1992 onwards was set to capture evidence from the last 28 years and the searches were limited to the English language. Searches were conducted without a study design filter. Bibliography searches of key papers were performed. The search strategies for each database can be found in Appendix 1.

### Article selection

A comprehensive and iterative approach to the literature searches for evidence was taken to ensure that a broad  range of perspectives was captured. Articles were included in the review if the following criteria were met: (1) used an adult study population and (2) compared risk of progression [e.g., (decline in) (estimated) glomerular filtration rate (GFR)] and/or mortality for ≥2 ethnic groups with pre-dialysis CKD. Articles that did not compare outcomes between ethnic minority and non-ethnic minority groups or focused on dialysis/transplant patients were excluded. Two reviewers (HH and RH) reviewed articles to determine eligibility for inclusion. Any discrepancies were resolved by discussion with a third reviewer (SF).

### Data extraction and synthesis

Data were extracted into a series of evidence tables, developed a priori, by one reviewer (HH). One evidence table was produced for each outcome group. Each table included details on the authors, date of publication, country in which the study was conducted, study aims, ethnic groups included in the study, study design, outcomes of interest, and key findings on factors associated with any ethnic differences in CKD outcomes for each study. Evidence tables can be found in Appendix 2. A narrative synthesis approach was taken to summarise the evidence.

## Results

### Search strategy, study selection and data extraction

**Fig. 1:**
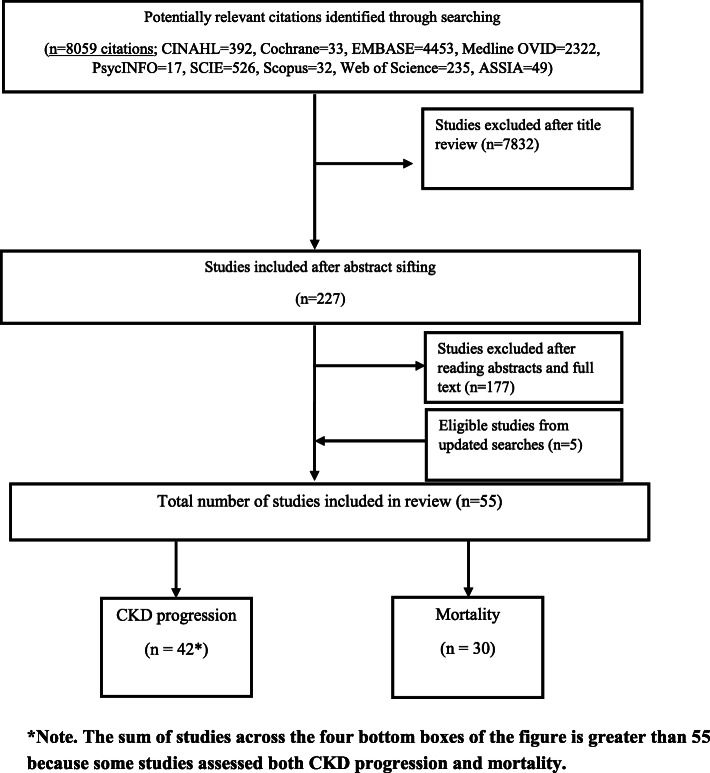
Flow diagram of searching and selection process

The results of the search strategy and selection process are shown in Fig. 1. 8059 citations were identified from the search. After removing duplicates and title and abstract screening 227 studies met the criteria for full text review, from which 50 were selected for inclusion in the review. Our updated searches identified 5 relevant studies published between July 2017 and February 2020, resulting in a total of 55 studies included in the review. These 55 studies included 1 systematic review [9] and 8 literature reviews on CKD progression [12–19]. Together these reviews included 19 of the individual studies identified in our searches. The reviews were published between 2004 and 2018, addressed slightly different research questions, and included different studies (Table 1). Only two of the reviews [9, 19] used a systematic search strategy and reported clear inclusion/exclusion criteria. Forty-two studies (*n* = 15,204,453 individuals; median: 3785, IQR: 1208-25,774) examined differences in CKD progression and thirty studies (*n* = 4,480,316 individuals, median: 3939, IQR: 1798-22,634) assessed ethnic differences in survival.

**Table 1:** Summary of previous reviews on ethnic differences in CKD progression and/or mortality

Review	Date	Number of studies included	Question/ Objectives	Study design	Ethnic groups	Inclusion/Exclusion criteria	What it answered	Studies included
Cass et al	2004	189	Explores the linkages between disadvantage, often accompanied by geographic isolation, and both the initiation of renal disease, and its progression to end stage renal disease (ESRD).	Discussion paper	Indigenous, non-Indigenous Australians	Unclear	Primary renal disease, genetic factors, early development and socioeconomic factors might explain excess burden of renal disease in indigenous populations	Weiner et al. (2004), Hsu et al.(2003), Mehrotra et al. (2008), Newsome et al. (2006)
Powe et al	2005	75	Summarizes work that has been done to understand the reasons for a higher burden of CKD in racial and ethnic minorities and indicates where more focus needs to be placed, thereby providing a framework for the goal of prevention of CKD and its progression in these high-risk groups	Non-systematic literature review	African-Americans, Native Americans, Hispanics, Caucasians	Unclear	Ethnic minorities make up a disproportionate share of the ESRD population in the United States. Reasons for this are multifactorial including a concentration of biologic-clinical, sociodemographic, and behavioural risk factors for CKD among certain racial and ethnic minorities. Behavioural factors including patient and provider interactions are not yet fully explored and may be central to the delivery of optimal care and prevention of ESRD in racial and ethnic minorities.	
Norris et al	2008	7	Commentary on Newsome et al. (2008) paper and considers key issues around CKD risk factors to better understand racial differences in rates of end stage renal disease	Review/ commentary on published paper	Caucasians, African-Americans, Asians, American Indians/Alaska Natives, and Hispanics	Unclear	Biological factors (e.g.: genes) and environmental influences are associated with CKD progression.	Newsome et al. (2006), Weiner et al. (2004), Hsu et al. (2003), Mehrotra et al. (2008)
Barbour et al.	2010	5 (and additional 8 discussed)	Summarizes the available evidence on ethnic differences in the rates of CKD progression towards ESRD	Systematic review	Caucasians, African-Americans, Afro-Carribbeans, Hispanics	Studies that directly observe rates of GFR decline in CKD cohorts of different races	The available evidence to date does not conclusively support the hypothesis of ethnic differences in the rates of progression through all-cause CKD. There are few properly designed studies that address this issue, and several often-cited studies have some methodological shortcomings that make interpretation difficult.	Choi et al. (2009), Hsu et al. (2003), Peralta et al. (2006)
Crews et al.	2014	33	Reviews studies exploring ethnic and socioeconomic disparities in CKD	Literature review	African-Americans, Caucasians	Studies on disparities of CKD	Geographic disparities in CKD prevalence, progression and treatment exist. CKD progression is more rapid for ethnic minority groups as compared to whites and may be largely, but not completely explained by genetic factors.	Van den Beukel et al. (2013), Derose et al. (2013), Kovesdy et al. (2013), Samuel et al. (2014)
Horowitz	2015		Explores the ethnic disparities in the prevalence, treatment, risks and outcomes of hypertension in patients with CKD.	Discussion paper	Caucasians, Hispanics, African-Americans	Unclear	Control of BP in patients at all stages of CKD remains suboptimal.	Hsu et al. (2005), Hebert et al. (1997); Hsu et al. (2006)
Harding et al	2017	48	Discusses genetic and social determinants of CKD in African-Americans and the impact of late referrals from primary care physicians to nephrologists on CKD outcomes	Literature review	Caucasians, African Americans	Unclear	Several factors contribute to disparities in outcomes for African Americans compared to Caucasians, including genetic and social determinants, late referrals, poor care coordination, medication adherence, low recruitment in trials.	
Jadawi et al	2018	32	Assesses the difference in the prevalence and progression of diabetic nephropathy, and the development of ESRD in people from three different ethnic groups with type 2 diabetes	Systematic review and meta-analysis	Caucasian, South Asian, African-Americans/Afro-Caribbeans	Studies comparing Caucasian, South Asian and African Caribbean, in whichever combination, in adult patients with T2DM and diabetic nephropathy	There was no significant link between ethnicity (South Asian, Caucasian and African Caribbean) and the prevalence of microalbuminuria; however, the pooled incidence rate ratio for ESRD in African Caribbean compared with Caucasian participants was significantly higher. Further research is needed to explore the potential non-albuminuric pathways of progression to ESRD	Earle et al., (2001), Koppiker et al. (1998), Ali et al. (2013), Mathur et al. (2018), Lewis et al. (2015)
Chen	2018	10	Commentary on Crews et al. (2018) paper and considers confounders of the association between dietary acid load and CKD progression	Review/ commentary on published paper	Caucasians, African Americans	Unclear	Racial disparities in the relation between dietary acid load and risk of ESRD may be confounded by severity, control, duration of diabetes and hypertension, and antihypertensive medications.	Crews et al. (2018), Parsa et al. (2013)

### Ethnic differences in CKD progression

#### African-Americans and Afro-Caribbean ethnicity

Thirty-six studies explored CKD progression for African-Americans or Afro-Caribbeans and Caucasians (Table 2). Studies were conducted in the US, Canada, UK, and Norway and most were prospective or retrospective cohort studies. Overall, most (*n* = 24) studies reported higher risk of CKD progression in African-American/Afro-Caribbean (adjusted (for varying covariates) HRs ranging in 15 studies from 1.16 (95% CI: 1.09–2.62) to 4.00 (95% CI: 2.99–5.35)) [13–16, 19–38]. However, some (*n* = 12) studies found no significant ethnic differences in risk of CKD progression, which may be partly due to smaller study sample size, duration of follow up, and/or adjustment for confounders and mediators [9, 38–48]. Studies adjusted for demographics factors (such as age and sex, with fewer studies adjusting for socioeconomic status), biological factors (such as baseline estimated glomerular filtration (eGFR) levels, proteinuria, baseline creatinine, cholesterol, haemoglobin, blood pressure), comorbidities (hypertension, diabetes, cardiovascular disease). Some studies also adjusted for body mass index, smoking, and prescribed medication.

**Table 2:** Summary of studies on ethnic differences in CKD progression

Comparison group (number of studies)	Country of study	Number of studies	Studies	Study design	Finding	Summary findings
African-American/Afro-Caribbean (AA) vs Caucasian (*n* = 36)	US	29	Agarwal et al., 2008	Prospective cohort study	Higher risk for AA	24 studies reported higher risk for AA, 12 studies found no significant differences
			Alves et al., 2010	Retrospective cohort study	Higher risk for AA	
			Babayev et al., 2013	Prospective cohort study	Higher risk for AA	
			Chen et al., 2018	Commentary	Higher risk for AA	
			Choi et al., 2009	Prospective cohort study	Higher risk for AA	
			Crews et al., 2014	Literature review	Higher risk for AA	
			Crews et al., 2018	Prospective cohort study	Higher risk for AA	
			Derose et al., 2013	Retrospective cohort study	Higher risk for AA	
			Fischer et al., 2016	Prospective cohort study	Similar risk	
			Grams et al., 2017	Prospective cohort study	Similar risk	
			Go et al., 2018	Retrospective cohort study	Similar risk	
			Hall et al., 2010	Prospective cohort study	Higher risk for AA	
			Harding et al., 2017	Literature review	Higher risk for AA	
			Hebert et al., 1997	Randomized clinical trial	Similar risk	
			Horowitz et al., 2015	Literature review	Higher risk for AA	
			Hsu et al., 2003	Birth cohort analysis	Higher risk for AA	
			Hsu et al., 2005	Prospective cohort study	Higher risk for AA	
			Hunsicker et al., 1997	Randomized prospective trial	Higher risk for AA	
			Jawadi et al., 2018	Systematic review and meta-analysis	Higher risk for AA	
			Jolly et al., 2014	Prospective cohort study	Similar risk	
			Jones-Burton et al., 2005	Prospective cohort study	Higher risk for AA	
			Kovesdy et al., 2009	Prospective cohort study	Similar risk	
			Lewis et al., 2015	Randomized, double-blind, placebo-controlled study	Higher risk for AA	
			Lucas et al., 2008	Prospective cohort study	Higher risk for AA	
			Menon et al., 2008	Retrospective cohort study	Similar risk	
			Norris et al., 2008	Commentary/review	Higher risk for AA	
			Parsa et al., 2013	Prospective cohort study	Higher risk for AA	
			Salifu et al., 2009	Prospective cohort study	Similar risk	
			Yang et al., 2014	Prospective cohort study	Higher risk for AA	
	Canada	1	Barbour et al., 2010	Systematic review	Similar risk	
	UK	5	Ali et al., 2013	Prospective cohort study	Similar risk	
			Dreyer et al., 2013	Retrospective cohort study	Higher risk for AA	
			Earle et al., 2001	Retrospective case-note review	Similar risk	
			Hull et al., 2011	Cross-sectional study	Similar risk	
			Mathur et al., 2018	Prospective cohort study with nested case-control study	Higher risk for AA	
	Norway	1	Van den Beukel et al., 2013	Prospective cohort study	Higher risk for AA	
South Asian (SA) (n = 12)	US	4	Hall et al., 2010	Prospective cohort study	Higher risk for SA	6 studies reported similar risk for SA compared to Caucasians, 6 reported higher risk for SA
			Derose et al., 2013	Retrospective cohort study	Higher risk for SA	
			Lewis et al., 2015	Randomized, double-blind, placebo-controlled study	Similar risk	
			Jawadi et al., 2018	Systematic review and meta-analysis	Similar risk	
	Canada	1	Barbour et al., 2010	Prospective cohort study	Similar risk	
	UK	7	Ali et al., 2013	Prospective cohort study	Similar risk	
			Dreyer et al., 2013	Retrospective cohort study	Higher risk for SA	
			Earle et al., 2001	Retrospective case-note review	Higher risk for SA	
			Hull et al., 2011	Cross-sectional study	Higher risk for SA	
			Koppiker et al., 1998	Retrospective case review	Similar risk	
			Mathur et al., 2018	Prospective cohort study with nested case-control study	Higher risk for SA	
			Pallayova et al., 2015	Prospective cohort study	Similar risk	
East Asian (EA) (n = 4)	US	3	Hall et al., 2010	Prospective cohort study	Higher risk for EA	3 studies reported higher risk for EA, 1 reported no significant differences
			Derose et al., 2013	Retrospective cohort study	Higher risk for EA	
			Lewis et al., 2015	Randomized, double-blind, placebo-controlled study	Similar risk	
	Canada	1	Barbour et al., 2010	Prospective cohort study	Higher risk for EA	
Asian - Pacific Islander (PI) (n = 4)	US	4	Derose et al., 2013	Retrospective cohort study	Higher risk for PI	2 studies reported higher risk for PI, 2 reported no significant differences
			Go et al., 2018	Retrospective cohort study	Similar risk	
			Hall et al., 2010	Prospective cohort study	Higher risk for PI	
			Lewis et al., 2015	Randomized, double-blind, placebo-controlled study	Similar risk	
Hispanic (*n* = 7)	US	6	Derose et al., 2013	Retrospective cohort study	Higher risk for Hispanics	6 studies reported higher risk for Hispanics, 1 reported similar risk
			Fischer et al., 2016	Prospective cohort study	Higher risk for Hispanics	
			Hall et al., 2010	Prospective cohort study	Higher risk for Hispanics	
			Horowitz et al., 2015	Literature review	Higher risk for Hispanics	
			Lewis et al., 2015	Randomized, double-blind, placebo-controlled study	Higher risk for Hispanics	
			Peralta et al., 2006	Prospective cohort study	Higher risk for Hispanics	
	Canada	1	Barbour et al., 2010	Systematic review	Similar risk	
Native American (n = 2)	US	2	Go et al., 2018	Retrospective cohort study	Similar risk	
			Lewis et al., 2015	Randomized, double-blind, placebo-controlled study	Similar risk	2 studies reported similar risk
Indigenous populations (n = 2)	Australia	1	Cass et al., 2004	Discussion paper	Higher risk for indigenous populations	Both studies reported higher risk
	Canada	1	Samuel et al., 2014	Prospective cohort study	Higher risk for indigenous populations	

Lower baseline eGFR levels [23, 25, 34], proteinuria [34, 35],  albuminuria [26]^,^ and higher (treated) blood pressure [31, 41, 48], and glycaemic control [45, 47] predicted increased risk of progression in African-Americans compared to Caucasians. Existing diabetes, cardiovascular diseases and congestive heart failure either fully or partially attenuated the association of increased risk of progression observed for African-Americans [26, 31, 43] compared to Caucasians. Apolipoprotein E and variants in the gene encoding apolipoprotein L1 (APOL1) explained some differences in progression for African-Americans compared to Caucasians [28, 33]. For example, there was a mean adjusted difference in eGFR slope of − 1.05 ml per minute per 1.73m^2^ per year (*p* < 0.001) for African-Americans in the high risk APOL1 group compared to Caucasians but no significant difference between rate of eGFR decline for African-Americans in the low risk APOL1 group and Caucasians [33].

#### South Asians

Twelve studies explored CKD progression in South Asians [18, 25, 26, 31, 35, 36, 46–51]. These studies were conducted in the US, Canada, and UK. Most were prospective or retrospective studies. Six studies reported similar risk of progression for South Asians compared to Caucasians and 6 reported higher risk for South Asians (for example 2 studies reported odds ratios (95%CI) of 1.44 (1.00–1.85) and 1.41 (1.32–1.51). Studies adjusted for demographic, biological factors, comorbidities, smoking, and prescribed medication and indicated eGFR levels [25], proteinuria [34, 35], higher (treated) blood pressure [48], and renal comorbidities [49] may explain these ethnic differences.

#### East Asians

Four studies explored CKD progression in East Asians, of which 3 were conducted in the US and 1 in Canada. Three were cohort studies and 1 was a randomised controlled trial (RCT). Three studies found higher risk for East Asians (one study reporting adjusted OR (95% CI): 1.41 (1.32–1.51)) and 1 study found similar risk of progression for East Asians compared to Caucasians [25, 26, 31, 49]. Studies adjusted for demographic, biological and comorbid factors. There was some evidence that renal comorbidities [31, 49] may contribute to South Asians having higher risk of CKD progression that Caucasians.

#### Pacific islanders

Four US studies included Pacific Islanders [25, 26, 31, 40]. One was a prospective cohort study, 2 retrospective cohort studies, and 1 RCT. Two studies reported higher risk of CKD progression for Pacific Islanders - 1 study reporting adjusted OR (95% CI): 1.41 (1.32–1.51) and another study reporting adjusted HR (95% CI): 3.84  (2.73–5.40), and 1 study found no significant differences in CKD progression for Pacific Islanders compared to Caucasians (adjusted OR (95% CI): 1.02 (0.91–1.15)). Factors contributing to ethnic differences in CKD progression for Pacific Islanders compared to Caucasians were unclear/unexplored.

#### Hispanics

Seven studies compared CKD progression for Hispanics and Caucasians. Six were conducted in the UK and 1 in Canada. Four were prospective or retrospective cohort studies, 2 were  reviews and 1 was an RCT. Six studies reported higher risk of CKD progression for Hispanics compared to Caucasians (effect estimates ranging from adjusted HR (95% CI): 1.93 (1.72–2.17) to HR (95% CI): 2.20 (1.46–3.30), and adjusted OR (95% CI): 1.49 (1.42–1.56)) [15, 25, 26, 31, 38, 52]. One study reported similar risk [9]. Studies suggested eGFR levels [25, 31], albuminuria [26], body mass index [31], blood pressure [31], diabetes [38], and prior cardiovascular disease [31] may explain the higher risk of CKD progression observed for Hispanics.

#### Native Americans

Two US studies explored CKD progression for Native Americans: 1 retrospective cohort study and 1 RCT. Both studies reported similar risk for CKD progression for Native Americans compared to Caucasians (for example, Go et al. (2018) reported adjusted OR (95% CI):1.57 (0.81–3.04)) [31, 40].

#### Indigenous populations

Two studies, 1 conducted in Australia and 1 in Canada, focused on CKD progression in Indigenous populations [17, 53]. One was a discussion paper and the other a prospective cohort study. Both studies reported higher risk of CKD progression for Indigenous populations compared to Caucasians. Samuel et al. (2014) reported adjusted HR (95% CI): 18.67 (10.77–32.36) vs 6.33 (5.41–7.40) for First Nation vs non First Nation individuals, respectively. The discussion paper explored pathways through which socioeconomic factors explained these differences.

### Ethnic disparities in all-cause mortality

#### African-Americans and afro-Caribbeans

Twenty-seven studies examined mortality differences for African-American or Afro-Caribbeans compared to Caucasians (Table 3). Most were conducted in the US and were either prospective or retrospective cohort studies. Eighteen studies reported no significant ethnic differences in survival [20–22, 26, 33, 34, 36, 38, 39, 42, 44, 46, 54–59]. Five studies found higher risk of mortality for African-Americans [13, 23, 60–62] (adjusted HRs (95% CI) ranging in studies from 1.30 (1.02–1.65) to 1.83 (1.33–2.52)), and 4 reported lower risk of mortality for African-Americans [25, 43, 63, 64] (adjusted HRs (95% CI) ranging in studies from 0.67 (0.63–0.72) to 0.79 (0.61–0.97)). Studies suggested age [25, 63], biological factors (e.g., higher blood pressure, serum albumin), comorbidities (cardiovascular disease, diabetes) and medication use (e.g.; more frequent use of calcitriol and less use of statins in African-Americans compared to Caucasians) [43, 63] may partly explain ethnic differences in survival for African-Americans/Afro-Caribbeans and Caucasians with CKD. There were mixed findings on the role of socioeconomic status in explaining these differences [60, 61].

**Table 3:** Summary of studies on ethnic differences in mortality for individuals with CKD

Comparison group (number of studies)	Country of study	Number of studies	Studies	Study design	Finding	Summary findings
African-American/Afro-Caribbean (AA) vs Caucasian (*n* = 27)	US	24	Agarwal et al., 2008	Prospective cohort study	Similar risk	5 studies reported higher risk for AA, 4 studies reported lower risk, 18 studies found no significant differences
			Alves et al., 2010	Retrospective cohort study	Similar risk	
			Babayev et al., 2013	Prospective cohort study	Similar risk	
			Cardarelli et al., 2008	Prospective cohort study	Similar risk	
			Choi et al., 2009	Prospective cohort study	Higher risk for AA	
			Derose et al., 2013	Retrospective cohort study	Lower risk for AA	
			Fedewa et al., 2014	Prospective cohort study	Higher risk for AA	
			Fischer et al., 2016	Prospective cohort study	Similar risk	
			Grams et al., 2017	Prospective cohort study	Similar risk	
			Hall et al., 2010	Prospective cohort study	Similar risk	
			Hayes et al., 2012	Prospective cohort study	Similar risk	
			Jolly et al., 2011	Retrospective cohort study	Similar risk	
			Jolly et al., 2014	Prospective cohort study	Similar risk	
			Kovesdy et al., 2009	Prospective cohort study	Lower risk for AA	
			Kovesdy et al., 2013	Historical cohort	Lower risk for AA	
			Mehrotra et al., 2008	Prospective cohort study	Higher risk for AA	
			Menon et al., 2008	Retrospective cohort study	Similar risk	
			Navaneethan et al., 2011	Retrospective cohort study	Similar risk	
			Newsome et al., 2006	Retrospective cohort study	Lower risk for AA	
			Norris et al., 2008	Commentary/review	Higher risk for AA	
			Parsa et al., 2013	Prospective cohort study	Similar risk	
			Weiner et al., 2004	Retrospective cohort study	Higher risk for AA	
			Wetmore et al., 2011	Prospective cohort study	Similar risk	
			Yang et al., 2014	Prospective cohort study	Similar risk	
	UK	3	Ali et al., 2013	Prospective cohort study	Similar risk	
			Hutchison et al., 2014	Prospective cohort study	Similar risk	
			Mathur et al., 2018	Prospective cohort study with nested case-control study	Similar risk	
South Asian (SA) (*n* = 8)	US	3	Derose et al., 2013	Retrospective cohort study	Lower risk for SA	6 studies reported lower risk for SA compared to Caucasians, 2 reported no significant differences
			Hall et al., 2010	Prospective cohort study	Lower risk for SA	
			Jolly et al., 2011	Retrospective cohort study	Lower risk for SA	
	Canada	2	Barbour et al., 2010	Prospective cohort study	Similar risk	
			Conley et al., 2012	Prospective cohort study	Lower risk for SA	
	UK	3	Ali et al., 2013	Prospective cohort study	Similar risk	
			Hutchison et al., 2014	Prospective cohort study	Lower risk for SA	
			Mathur et al., 2018	Prospective cohort study with nested case-control study	Lower risk for SA	
East Asian (EA) (*n* = 5)	US	3	Derose et al., 2013	Retrospective cohort study	Lower risk for EA	All studies reported lower risk for EA
			Hall et al., 2010	Prospective cohort study	Lower risk	
			Jolly et al., 2011	Retrospective cohort study	Lower risk for EA	
	Canada	2	Barbour et al., 2010	Prospective cohort study	Lower risk for EA	
			Conley et al., 2012	Prospective cohort study	Lower risk for EA	
Asian - Pacific Islander (PI) (n = 3)	US	3	Derose et al., 2013	Retrospective cohort study	Lower risk for PI	2 studies reported lower risk for PI, 1 reported no significant differences
			Hall et al., 2010	Prospective cohort study	Similar risk	
			Jolly et al., 2011	Retrospective cohort study	Lower risk for PI	
Hispanic (*n* = 6)	US	6	Derose et al., 2013	Retrospective cohort study	Lower risk for Hispanics	3 studies reported lower risk for Hispanics, 3 studies reported similar risk
			Fischer et al., 2016	Prospective cohort study	Similar risk	
			Hall et al., 2010	Prospective cohort study	Similar risk	
			Jolly et al., 2011	Retrospective cohort study	Lower risk for Hispanics	
			Peralta et al., 2006	Prospective cohort study	Lower risk for Hispanics	
			Mehrotra et al., 2008	Prospective cohort study	Similar risk	
Native American (n = 1)	US	1	Jolly et al., 2011	Retrospective cohort study	Higher risk for Natives	1 study reported higher risk for Native Americans

#### South Asians

Eight studies explored ethnic differences in mortality for South Asians compared to Caucasians. Studies were conducted in the US (*n* = 4), Canada (*n* = 2), and UK (*n* = 2). All were either retrospective or prospective cohort studies. Six reported lower risk of mortality for South Asians compared to Caucasians (adjusted HR (95% CI) ranging from 0.33 (0.14–0.64) to 0.73 (0.59–0.88)), and 2 reported no significant differences (e.g., OR (95% CI): − 0.51 (− 3.25 to − 2.23)) [33, 34, 36, 46, 49, 56, 59, 65]. Studies that reported lower risk of mortality for South Asians suggested age and sex, proteinuria, blood pressure, diabetes, cardiovascular disease and medications (antihypertensive drugs and statins), and C-reactive protein partly explained ethnic differences in mortality [49, 59].

#### East Asians

Five cohort studies – 3 conducted in the US and 2 in Canada- explored mortality differences for East Asians and Caucasians. All 5 studies reported lower risk of mortality for East Asians compared to Caucasians (adjusted HR (95% CI) ranging from 0.58 (0.52–0.65) to 0.69 (0.55–0.88)) [25, 26, 49, 56, 65]. Age and sex, proteinuria, blood pressure, diabetes, cardiovascular disease and medications (antihypertensive drugs and statins), and C-reactive protein [49, 59] partly explained the lower risk of mortality observed for East Asians.

#### Pacific islanders

Three US studies, 1 prospective and 2 retrospective cohort studies, explored mortality differences for Asians/Pacific Islanders and Caucasians [25, 26, 56]. Two reported lower risk of mortality for Pacific Islanders (adjusted HR (95% CI) ranging from 0.58 (0.52–0.65) to 0.76 (0.61–0.95) and 1 reported no significant differences. Factors contributing to ethnic differences in mortality for Pacific Islanders compared to Caucasians were not fully explored.

#### Hispanics

Six US studies examined ethnic differences in mortality for Hispanics and Caucasians [25, 26, 38, 52, 56, 61]. Studies were either prospective or retrospective cohort studies. Three studies reported lower risk of mortality for Hispanics compared to Caucasians (adjusted HR (95% CI) ranging from 0.66 (0.50–0.94) to 0.72 (0.66–0.79)) and 3 reported similar risk (adjusted HR (95% CI) ranging from 0.79 (0.59–1.35) to 0.94 (0.74–1.20)). Lower risk of mortality observed for Hispanics was partly explained by differences in urine protein levels [38] (with Hispanics having significantly lower risk of mortality than Caucasians at higher levels of urine protein but no significant ethnic differences at lower levels of urine protein) and not explained by differences in hypertension, diabetes, and use of medication including insulin [52].

#### Native Americans

One US retrospective cohort study compared mortality differences for Native Americans and Caucasians and reported higher risk of mortality for Native Americans (adjusted HR (95% CI):1.41 (1.08–1.84)) [56]. Factors contributing to mortality differences for Native Americans compared to Caucasians were not clear.

## Discussion

We used an established and systematic methodology and searched a range of databases to capture the full range of existing studies on ethnic differences in pre-dialysis CKD progression and mortality. This scoping review identified evidence for higher risk of CKD progression in Afro-Caribbean/African-Americans, Hispanics, Asians compared to Caucasians which was at least partly explained by biological factors (e.g.: blood pressure) and comorbidities (such as diabetes, and cardiovascular disease), and lower risk of mortality for South and East Asians and Pacific Islanders compared to Caucasians. Our scoping review also identified mixed findings on risk of mortality for African-Americans and Hispanics compared to Caucasians. Future studies need to explore this, as studies reporting significant findings did not differ in the range of adjusted confounders compared to studies that found significant differences. The role of medication in the association between ethnicity and progression and mortality is complex, as differences in medication may represent unmet need in certain ethnic groups or may be an indicator of disease severity in individuals.

### Gaps in the literature

A key gap in this literature is understanding why Asians (South, East, and Pacific Islanders) and Hispanics live longer, despite having higher prevalence of comorbidities such as diabetes, cardiovascular disease, and heart failure. Future research may explore potential missing factors that may explain why these groups experience increased risk CKD progression but live longer. The search also identified most research on ethnic differences in CKD progression and mortality has been conducted in US populations and there is less research in other countries with a significant proportion of ethnic minority immigrant populations (e.g.: UK, Canada), as well as lower/middle income countries. Across all studies, comparisons were made mostly between Caucasians vs. African-Americans and Caucasians vs. Hispanic (in US studies), and Caucasians vs. Afro-Caribbean and Caucasian vs. South Asian (in UK studies). Fewer studies compared Caucasians to East Asians, Pacific Islanders or Native Americans. Furthermore, most studies did not distinguish between subgroups within an ethnic group (e.g.: Africans vs. Caribbean in the same ethnic group). There may be heterogeneity in findings within ethnic groups, as shown in a recent UK study that found risk of CKD progression was higher in Bangladeshis compared to Indians [36]. Similarly, identified studies did not distinguish or adjust for generational status or indigenous vs immigrant populations, though some existing studies may not have been captured through our searches.

Some studies did not adjust for important confounders of the association between ethnicity and CKD outcomes. Firstly, very few (*n* = 15) of the included studies adjusted for socioeconomic status. This is particularly important for US studies, where low socioeconomic status is closely linked to ethnicity and independently associated with health insurance and access to health care. Secondly, most studies on CKD progression did not account for competing risk of death, so differences in progression in these studies may have been at least partly due to differences in survival across ethnic groups. A limited number of studies (*n* = 18) assessed ethnic differences in both progression and mortality. These studies seemed to suggest Hispanics and East and South Asians experience increased risk of CKD progression as result of lower competing risk of death, and poorer evidence for significant differences in risk of mortality for African-Americans and Hispanics compared to Caucasians. However, further research is needed to confirm these findings. Thirdly, there were few studies exploring genetic risk factors for CKD progression and mortality. Some studies have suggested other genetic risk factors such as genes encoding non-muscle myosin heavy chain type II isoform A for ESKD in African-Americans [66], but there is a lack of genetic studies comparing risk of ESKD across different ethnic groups with pre-dialysis CKD. Future studies may also explore whether differences between ethnic groups hold across countries or whether they differ due to societal and health care reforms. Fourthly, some studies did not assess progression and mortality stratified by level of CKD severity, making it difficult to directly compare or identify if ethnic differences in progression and mortality vary at different stages of CKD. An updated systematic review and meta-analysis that goes beyond scoping to assess bias in these studies and pool together estimates from the different studies (where possible) may help explain some of the mixed findings and improve our understanding of the extent and key predictors of ethnic differences in CKD outcomes. Finally, there was lack of data on level of control of biologic factors such as blood pressure and glycemia, as well as limited data on medication and adherence, and how these vary across ethnic groups, all of which are important for differences in CKD progression and mortality. Future studies should aim to capture this data as much as possible.

### Limitations

The review was based on a comprehensive search of the literature. However, it is possible that some relevant studies may have been missed as the search was restricted to studies that were published in English and published after 1992. Studies that examined CKD progression and outcomes for only one ethnic group and did not make comparisons with another ethnic group were also excluded. Furthermore, there was limited additional searching of grey literature, though we believe the majority of relevant studies will have been captured through the different databases and bibliography.

An important limitation of the scoping review approach is that papers are not critically appraised in detail and quality of the individual studies is therefore not assessed [10]. However, this scoping review was based on established methodology [10] and has mapped the existing literature on ethnic differences in CKD outcomes, identified gaps in the research and highlighted the need for further systematic reviews and additional primary research focusing on cardiovascular-related and other adverse outcomes for pre-dialysis CKD.

### Clinical and policy implications of this scoping review

Increased risk of CKD progression in ethnic minority groups may be tackled through closer monitoring and management of renal comorbidities such as diabetes and cardiovascular disease, for example through proteinuria and blood pressure measurement, particularly in these high risk groups. There has been some evidence to suggest incentivisation programs, such as the Quality and Outcomes Framework programme in the UK, may help improve care for diabetics with CKD [67]. Interventions including the use of medications such as renin-angiotensin-aldosterone system blockers and patient-provider education interventions may also reduce risk of progression in high-risk groups [68]. In the UK, a national quality improvement programme has mapped laboratory data taken from all settings to derive graphs of kidney function over time. Declining kidney function is then flagged by a laboratory scientist and sent to primary care doctor for clinical review and referral, where necessary [69]. However, a better understanding of risk factors for CKD progression in high risk groups is needed to help develop more effective and targeted interventions.

## Conclusions

Scoping reviews are a relatively novel method of systematically assessing a wide range of literature in a particular field, in order to identify important gaps in the literature, and inform more targeted systematic reviews or further studies. This is the first synthesis of the extensive body of literatures on ethnic differences in CKD progression and mortality. The findings of this review suggest higher rates of RRT in ethnic minority groups may be partly due to increased risk of progression and reduced mortality in these groups (compared to Caucasians), though more evidence is needed for African-American and Hispanic ethnicity. The review highlights the need for further studies using similar approaches and adjusting for the same confounders, which would improve our understanding of disease progression and mortality in people with CKD.

## Supplementary information

**Additional file 1: Appendix 1.** Search strategies. **Appendix 2**. Evidence tables.

## Data Availability

Not applicable.

## Abbreviations

APOL 1: Apolipoprotein L1
CKD: Chronic kidney disease
ESKD: End-stage kidney disease
GFR: Glomerular filtration rate
HIV: Human Immunodeficiency virus
HR: Hazard ratio
IQR: Interquartile range
RRT: Renal replacement therapy
UK: United Kingdom
US: United States
