# Supplementary material for: Ethnic minority disparities in progression and mortality of pre-dialysis chronic kidney disease: a systematic scoping review
Source: BMC Nephrol. 2020 Jun 9;21:217. doi: 10.1186/s12882-020-01852-3 (PMC7282112; doi:10.1186/s12882-020-01852-3)
Supplement: Supplementary file 1 — Additional file 1: Appendix 1. Search strategies. Appendix 2. Evidence tables. [file 12882_2020_1852_MOESM1_ESM.docx]

Appendix 1: Search strategies

All searches were run on the 6^th^ July 2017, except for Web of Knowledge search which was run on 14^th^ July 2017. Updated searches were run on 4^th^ February 2020.

***Medline OVID search strategy***

1 Kidney/

2 exp Kidney Diseases/

3 exp Renal Insufficiency/

4 exp Renal Dialysis/

5 exp Renal Replacement Therapy/

6 exp Proteinuria/

7 Glomerular Filtration Rate/

8 1 or 2 or 3 or 4 or 5 or 6 or 7

9 exp Ethnic Groups/

10 exp Continental Population Groups/

11 exp Minority Health

12 BME.mp.

13 bame.mp.

14 Racial.mp.

15 south Asian.mp.

16 Caucasian.mp.

17 9 or 10 or 11 or 12 or 13 or 14 or 15 or 16

18     Treatment Outcome/ or "Outcome Assessment (Health Care)"/ or outcome assessment.mp. or "Outcome and Process Assessment (Health Care)"/
19    outcome measure*.mp. [mp=title, abstract, original title, name of substance word, subject heading word, keyword heading word, protocol supplementary concept word, rare disease supplementary concept word, unique identifier, synonyms]
20     Quality of Life/
21     health impact assessment/
22     well-being.mp.

23     18 or 19 or 20 or 21 or 22
24     exp Mental Disorders/
25     exp Depression/
26     Anxiety/
27     Stress, Psychological/
28    exp Dementia/
29     exp Cardiovascular Diseases/
30     Myocardial Infarction/
31     exp Stroke/

32     exp mortality/

33 Survival/

34 Longevity/

35 Life expectancy/

36     24 or 25 or 26 or 27 or 28 or 29 or 30 or 31 or 32 or 33 or 34 or 35
37     exp research design/
38     exp empirical research/
39     exp qualitative research/
40     37 or 38 or 39
41     23 or 36
42     8 and 17 and 41 and 40

43 8 and 17 and 41

44 limit 43 to (yr="1992 -Current" and "all adult (19 plus years)" and english)

***CINAHL search strategy***

AB ( black or ethnic or race or ethnic minority or african american or hispanic or latino or latina or minority or african-american or colour or color or latino or mexican or spanish or puerto rican or asian or chinese or korean or japanese or asian american or indian or native or idigenous or aboriginal or first nation or white or Caucasian or continental population group ) AND AB ( kidney disease or renal disease or renal failure or kidney failure or nephropathy ) AND AB ( mortality or survival or longevity or life expectancy or morbidity or transplantation or quality of life or mental health or death or transplant or renal replacement therapy or outcome measure* or cardiovascular disease or myocardial infarction or stroke)

***EMBASE search strategy***

1. Kidney/

2. exp Kidney Diseases/

3. exp Renal Insufficiency/

4. exp Renal Dialysis/

5. exp Renal Replacement Therapy/

6. exp Proteinuria/

7. Glomerular Filtration Rate/

8. 1 or 2 or 3 or 4 or 5 or 6 or 7

9. exp Ethnic Groups/

10. exp Continental Population Groups/

11. exp Minority Health/

12. BME.mp.

13. bame.mp.

14. Racial.mp.

15. south Asian.mp.

16. Caucasian.mp.

17. 9 or 10 or 11 or 12 or 13 or 14 or 15 or 16

18. Treatment Outcome/ or "Outcome Assessment (Health Care)"/ or outcome assessment.mp. or "Outcome and Process Assessment (Health Care)"/

19. outcome measure*.mp.

20. "Quality of Life"/

21. health impact assessment/

22. well-being.mp.

23. 18 or 19 or 20 or 21 or 22

24. exp Mental Disorders/

25. exp Depression/

26. Anxiety/

27. Stress, Psychological/

28. exp Dementia/

29. exp Cardiovascular Diseases/

30. Myocardial Infarction/

31. exp Stroke/

32. exp mortality/

33. Survival/

34. Longevity/

35. Life expectancy/

36. 24 or 25 or 26 or 27 or 28 or 29 or 30 or 31 or 32 or 33 or 34 or 35

37. 23 or 36

38. 8 and 17 and 37

39. limit 38 to (english and yr="1992 -Current" and (adult <18 to 64 years> or aged <65+ years>))

***PsycINFO search strategy***

| 1. Treatment Outcome/ or "Outcome Assessment (Health Care)"/ or outcome assessment.mp. or "Outcome and Process Assessment (Health Care)"/ |  |
| --- | --- |
| 2. outcome measure*.mp. |  |
| 3. "Quality of Life"/ |  |
| 4. health impact assessment/ |  |
| 5. well-being.mp. |  |
| 6. 1 or 2 or 3 or 4 or 5 |  |

***Scopus search strategy***

TITLE-ABS-KEY ( *kidney*  AND *disease*  OR  *renal*  AND *disease*  OR  *renal*  AND *insufficiency*  OR  *kidney*  AND *failure* )  AND  TITLE-ABS-KEY ( *ethnic*  OR  *race*  OR  *ethnic*  AND *minority*  OR  *colour*  OR  *asian*  OR  *chinese*  OR  *native*  OR  *indigenous*  OR  *white*  OR  *caucasian*  OR  *first*  AND *nation*  OR  *african*  AND *american*  OR  *black*  OR  *mexican*  OR  *hispanic*  OR  *korean* )  AND  TITLE-ABS-KEY ( *morbidity*  OR  *survival*  OR  *longevity*  OR  *cardiovascular*  AND *disease*  OR  *mental*

AND *health*  OR  *renal*  AND *replacement*  AND *therapy*  OR  *dialysis*  OR  *transplant* ) )

***Applied Social Sciences Index & Abstracts (ASSIA)***

[ab(kidney disease OR renal disease OR renal insufficiency OR kidney failure) AND ab(ethnic OR race OR ethnic minority OR colour OR asian OR chinese OR native OR indigenous OR white OR caucasian OR first nation OR african american OR black OR mexican OR hispanic OR korean) AND ab(morbidity OR survival OR longevity OR cardiovascular disease OR mental health OR renal replacement therapy OR dialysis OR transplant) AND peer(yes) AND la.exact("English") AND pd(>19911231)](https://search-proquest-com.salford.idm.oclc.org/myresearch/savedsearches.checkdbssearchlink:rerunsearch/1255566/SavedSearches?site=assia&t:ac=SavedSearches)

***SCIE search strategy***

[ AbstractOmitNorms:'black or ethnic or race or ethnic minority or african american or hispanic or latino or latina or minority or african-american or colour or color or latino or mexican or spanish or puerto rican or asian or chinese or korean or japanese or asian american or indian or native or idigenous or aboriginal or first nation or white or Caucasian or continental population group'
 - AND AbstractOmitNorms:'kidney disease or renal disease or renal failure or kidney failure or nephropathy or renal insufficiency'
 - AND AbstractOmitNorms:'mortality or survival or longevity or life expectancy or morbidity or transplantation or quality of life or mental health or death or transplant or renal replacement therapy or outcome measure* or cardiovascular disease or myocardial infarction or stroke' ]

***Web of Knowledge Search strategy***

1. (TI=(kidney disease OR renal OR nephropathy or glomerular filtration rate OR proteinuria)) *AND* **LANGUAGE:** (English) *AND* **DOCUMENT TYPES:** (Article)
2. (TI= (ethnic* OR minority OR race OR racial OR African American OR black* OR white*)) *AND* **LANGUAGE:** (English) *AND* **DOCUMENT TYPES:** (Article)
3. (TI=(quality life OR mental health OR cardiovascular OR hypertension OR outcome OR progress* OR stroke OR myocardial infarction OR mortality OR survival)) *AND* **LANGUAGE:** (English) *AND* **DOCUMENT TYPES:** (Article)
4. #3 AND #2 AND #1

### Appendix 2: Evidence tables

**CKD progression**

| **Author & year** | **Country** | **Study aims** | **N** | **Study population** | **CKD stage/eGFR** | **Ethnic groups** | **Study design** | **Analysis** | **Outcomes** | **Adjusted confounders** | **Conclusions** |
| --- | --- | --- | --- | --- | --- | --- | --- | --- | --- | --- | --- |
| Agarwal et al., 2008 | US | To evaluate the competing risk of ESRD versus mortality and to evaluate the risk factors associated with these two outcomes in a CKD population | 220 | Patients at a Veterans Administration Medical Centre | eGFR <60ml/min/1.73m^2^ or urine protein/creatinine ration >0.22g/g | Caucasian, African American | Prospective cohort study | Competing risk Cox regression models | End-stage renal disease | Age, proteinuria, eGFR, systolic blood pressure, and coronary artery disease | African-American had higher risk of ESRD [adjusted HR (95% CI): 2.75 (1.09-6.92)] but similar risk of all-cause mortality [adjusted HR (95% CI): 1.15 (0.60-2.17)] |
| Ali et al., 2013 | UK | Compare the rate of progression of diabetic CKD in different ethnic groups | 329 | All new adult patients attending a tertiary renal unit in east London with diabetic chronic kidney disease between 2000 and 2007 and followed up till 2009 were included. Patients presenting with acute end-stage kidney failure were excluded. | No detail | South Asian, Caucasian, African-American | Prospective cohort study | linear regression | Annual decline in eGFR, progression to ESRD, and mortality in different ethnic groups | Baseline eGFR, systolic and diastolic blood pressure, proteinuria, ACE or angiotensin receptor blockers use, and glycated haemoglobin, ischemic heart disease, CVD, diabetic retinopathy | Annual decline in eGFR, numbers progressing to ESRD and total mortality not significant different across the 3 groups. Compared to Caucasians, adjusted mean annual decline was (OR:) -1.67 (-4.97 to 1.64) and -0.51 (-3.25 to 2.23) for African-Americans and South Asians, respectively. Risk of death for Afro-Caribbeans and South Asians compared to Caucasians was 1.01 (0.63-2.57) and 1.17 (0.68-2.32), respecitvely. |
| Alves et al., 2010 | US | Assess prevalence, incidence, rapid eGFR decline (≥50% decrease in baseline value), ESRD, and mortality among HIV-associated CKD | 2468 | HIV-infected adults from a non-profit clinic in Tennessee | eGFR <60ml/min/1.73m^2^ | Non-African-Americans and African-Americans | Retrospective cohort study | Mixed effects, competing risks, Poisson and Cox regression | eGFR decline, ESRD, mortality | Sex, race, baseline age, level of kidney function, anaemia, cardiovascular disease, absolute CD4 lymphocyte count (CD4), HIV-1 RNA viral load, history of angiotensin-converting enzyme inhibitor or angiotensin receptor blocker, HAART, opportunistic infection, hypertension (HTN), chronic hepatitis C, diabetes mellitus, and HIV risk group: men-having-sex-with-men, heterosexual contact, intravenous drug use, other, or unknown | Risk for rapid eGFR decline was similar for both groups. African Americans (AA) had significantly higher risk of ESRD and were more likely to die than non-African-Americans, although not statistically significant. For eGFR decline outcome, adjusted HR for AA compared to non-AA was 2.5 (0.9, 6.9). For death outcome, adjusted HR (95% CI) for AA compared to non-AA was 1.9 (0.6, 6.5) |
| Babayev et al., 2013 | US | Explore whether race and body mass index (BMI) influence end-stage renal disease (ESRD) and mortality rates in participants with CKD stages 3-4 | 14,631 | Individuals 18 years and older with high blood pressure, diabetes or a family history of kidney failure and CKD stage 3-4 (Kidney Early Evaluation Program participants) | CKD stage 3-4; eGFR: 15-59 mL/min/1.73 m^2^ | Caucasian, African-American | Prospective cohort study | Survival analyses model | End-stage renal disease, mortality | Age, sex, hypertension, diabetes, baseline eGFR, albuminuria | African-American race was associated with higher incidence of ESRD, but not death. In multivariable models, African American race increased the risk of ESRD (HR, 1.66; 95% CI, 1.26-2.07), but not death (HR, 0.89; 95% CI, 0.76-1.03). Male sex, hypertension, diabetes, lower baseline eGFR, and albuminuria were predictive of higher rates of ESRD. Age, male sex, diabetes, lower baseline eGFR and albuminuria were predictive of overall mortality. No significant interaction between race and BMI in progression to ESRD or death |
| Barbour et al., 2010 | Canada | Compare CKD progression across different ethnic groups | 3444 | CKD adult patients referred to nephrologist | eGFR < 60 mL/min/1.73 m2 alone or, if eGFR > 60 mL/min/1.73 m2, evidence of kidney damage from urinalysis or based on biopsy or ultrasound results | Caucasian, East Asian, South Asian | Prospective cohort study | Survival analyses (Cox proportional hazard analysis) | Time to death, renal replacement therapy, rate of decline in eGFR | Blood pressure, proteinuria, age, sex, diabetes, CVD, medications (ACEI, ARB, statins or vitamin D analogues) | Caucasians had higher risk of death (HR: 0.69 and 0.80, p<0.05) and slower rate of decline in eGFR (p=0.027) than South Asian or East Asian. East Asian and South Asian have higher risk of progressing to RRT and lower risk of death before RRT (HR: 0.72 and 0.52, p<0.05) compared to Caucasians) after adjusting for usual risk factors (age, sex, diabetes, cardiovascular disease (CVD),blood pressure, baseline eGFR levels and use of ACEI/ARB/vitamin D/statins). However, after adjusting for laboratory abnormalities (renal comorbidities), differences for progression were not significant, but survival advantage for Asians remained significant. |
| Barbour et al., 2010 | Canada | To summarise existing evidence on ethnic differences in the rates of CKD progression towards ESRD | 6090637 | Prospective cohort studies that directly observed the rates of renal progression in different ethnic groups with known all-cause CKD and cross-sectional studies | Any | Caucasian, African-American, Afro-Carribbean, Hispanic | Systematic review | n/a | CKD progression | Age, gender, socio-economic status, co-morbidities and known predictors of renal progression (proteinuria, hypertension, diabetes, baseline GFR and medication use), access to health services, blood pressure treatment | Available evidence up to 2010 did not support hypothesis of ethnic differences in the rates of progression through all-cause CKD |
| Cass et al., 2004 | Australia | Pathways leading from disadvantage to progression of CKD in indigenous and other Australians | review of studies | Indigenous Australians | No detail | Indigenous, non-Indigenous Australians | Discussion/review of proposed pathways of higher incidence ESRD in indigenous Australians relative to other Australians | n/a | ESRD |  | Age and sex adjusted incidence of ESRD is 9 times higher in indigenous Australians than non-indigenous Australians. This may be explained by different patterns in primary renal diseases, genetic factors, early development factors, socioeconomic factors. Almost half of new ESRD cases in indigenous Australians was attributed to diabetes compared to one in six non-indigenous cases. Glomerulonephritis was attributed as case of one in four indigenous and one in three non-indigenous patients. |
| Chen et al., 2018 | US | commentary on Crews et al (2018) paper and to consider confounders of the association between dietary acid load and CKD progression |  | CKD population | No detail | Caucasians, African Americans | commentary | n/a | CKD progression |  | Racial disparities in the relation between dietary acid load and risk of ESRD may be confounded by severity, control, duration of diabetes and hypertension, and antihypertensive medications. |
| Choi et al., 2009 | US | Compare risk of ESRD and death by level of eGFR at baseline | 2,015,891 | US veterans registered within the Department of Veterans Affairs health system | eGFR levels, 45-60, 3-44, 14-29, <30 | Caucasian, African-American | Prospective cohort study | Cox proportional hazard model | ESRD, mortality | Age, sex, baseline comorbidities, and socioeconomic status, delivery of healthcare (health centre), eGFR level | Risk of ESRD was higher for African-American compared to Caucasians at all levels of eGFR (including those <60ml/min/1.73m2). The adjusted hazard ratios (HR) for end-stage renal disease associated with African-American versus Caucasian for patients with an eGFR 45-59, 30-44, 15-29, and <15 mL/min/1.73m2, respectively were 3.08 (95% CI, 2.74-3.46), 2.47 (95% CI, 2.26-2.70), 1.86 (95% CI, 1.75-1.98), and 1.23 (95% CI, 1.12- 1.34). Age standardized rate of death was higher for African-American than Caucasians - association of African-American race with mortality was strongest at 45 ml/min/1.73m2 and was progressively attenuated at lower levels of eGFR. The highest risk of mortality associated with African-American ethnicity was also observed among those with an eGFR 45-59 mL/min/1.73m2 (HR 1.32, 95% CI, 1.27-1.36). |
| Crews et al., 2014 | US | Review of recent literature on ethnic disparities in CKD and identify opportunities for changes in policy and clinical practice | review of studies | Studies on disparities in CKD published in 2013 | Various stages of CKD | African-Americans, Caucasians | Literature review | n/a | CKD progression |  | Geographic disparities in CKD prevalence exist and vary by race. CKD progression is more rapid for racial and ethnic minority groups compared with whites and may be largely, but not completely, explained by genetic factors |
| Crews et al., 2018 | US | To determine the relation of dietary acid load to racial/ethnic differences in risk of ESRD among persons with CKD | 1123 | Non-institutionalized civilians (aged 20+ years) with CKD and who were not pregnant | eGFR ≥15 and <60 ml/min per 1.73 m^2^ | African-Americans, Caucasians | Prospective cohort study | Cox proportional hazards model | Development of ESRD | Net acid secretion, age, sex, poverty income ratio, body surface area, total caloric intake, serum bicarbonate, protein intake, diabetes, hypertension, estimated eGFR, urinary albumin-to-creatinine ratio | African-Americans had higher risk of ESRD (adjusted HR (95% CI): 1.68 (CI 1.18-2.38)) compared to Caucasians. A stronger association of net acid excretion (NAE) with risk of ESRD was observed among African-Americans (adjusted HR per mEq/d increase in NAE 1.21, CI 1.12-1.31) than among Caucasians (HR 1.08, CI 0.96-1.20), p interaction for race/ethnicity*NAEes=0.004. |
| Derose et al., 2013 | US | To examine racial differences in projected kidney failure (based on linear decline in eGFR rate) and mortality among those with projected kidney failure | 1,119,816 | Adult members of Kaiser Permanente Southern California from 2003–2009 with >2 serum creatinine tests and >180 days between tests | eGFR<60, 29-15, 30-44,45-59 | Hispanic, African-American, Asian (including Pacific Islanders), Caucasian | Retrospective cohort study | Linear regression (eGFR decline); Competing risk proportional hazard regression (mortality) | ESRD/eGFR decline, mortality | Age, sex, baseline eGFR | Projected kidney failure during CKD stage 3 and 4 was higher amongst African-Americans, Hispanics, and Asians compared to Caucasians. The ORs for projected kidney failure vs. Caucasians during CKD stages 3 and 4 were 1.54 (95% CI, 1.46– 1.62) in African-Americans, 1.49 (95% CI, 1.42–1.56) in Hispanics, and 1.41 (95% CI, 1.32–1.51) in Asians. Mortality among those with projected kidney failure was higher for Caucasians compared to African-Americans, Hispanics, Asians. Among those with projected kidney failure, the HRs of death vs. whites during CKD stages 3 and 4 were 0.82 (95% CI, 0.77–0.88) in African-Americans, 0.67 (95% CI, 0.63–0.72) in Hispanics, and 0.58 (95% CI, 0.52–0.65) in Asians. |
| Dreyer et al., 2013 | UK | Assess progression of CKD in a multi-ethnic cohort of people with diabetes managed in community settings | 3855 | All adults (aged between 30-75 years) with diabetes and registered with one of 134 general practices in east London | CKD stage 3a-5; eGFR between 16 to <60ml/min/1.73m^2^ | Caucasian, Afro-Caribbean, South Asian | Retrospective cohort study | Multi-level linear regression | CKD progression | eGFR, systolic and diastolic blood pressure, glycated haemoglobin, proteinuria, total cholesterol, smoking status, BMI, vascular co-morbidity (hypertension, ischaemic heart disease, heart failure, cerebrovascular disease) and prescription data for angiotensin-converting | Rate of CKD progression was significantly higher in South Asian (–1.01 ml/min/1.73 m^2^) compared to Caucasian groups (–1.01 ml min1 1.73 m^2^) (p=0.001). Among those with proteinuria at baseline, rate of CKD progression was faster for South Asians and Afro-Caribbean compared to Caucasians. |
| Earle et al., 2001 | UK | Assess variations in rate of progression of renal disease according to racial origin | 1684 | Adult attendees of a diabetic clinic | No detail | Indo-Asian, African-Caribbean, and Caucasian | Retrospective case-note review | Survival analyses | CKD progression | Systolic and diastolic blood pressure, glycaemic control, smoking habit, baseline proteinuria, haemoglobin, creatinine, and usage of angiotensin-converting enzyme inhibitors, age, gender | Proportion of patients doubling their creatinine was higher for Indo-Asians compared to African-Caribbean and Caucasians. Rate of renal function decline was higher for Indo-Asian compared to African-Caribbean and Caucasians; mean decline (95% CI) was 5.36 (2.21-8.52) for South Asians, compared to 3.14 (0.82-5.46) for African-Americans and 2.22 (1.31-3.14) for Caucasians (p=0.035). |
| Fischer et al., 2016 | US | Assess association between race, CKD progression, incident ESRD, mortality | 3785 | Adults with CKD | eGFR <60ml/min/1.73m^2^, <45 | Caucasian, African-American, Hispanic | Prospective cohort study | Cox proportional hazard analysis | CKD progression, ESRD | Age, sex, education, smoking, health insurance, received nephrology care at baseline, diabetes, systolic blood pressure, eGFR, 24 hour urine protein, body mass index, haemoglobin, ACEi/ARB use | Hispanics had significantly higher rates of CKD progression, incident ESRD, and mean annual decline in eGFR than did Caucasians (rate per 100 person years: 1.41 (p<0.001) but not African-Americans. In adjusted analyses, the risk of CKD progression did not differ between Hispanics and Caucasians or African-Americans. However, among nondiabetic participants, compared with NHB, Hispanics had a lower risk of CKD progression (HR: 0.61; 95% confidence interval, 0.39 to 0.95) and incident ESRD (HR: 0.50; 95% confidence interval, 0.30 to 0.84). African-American patients with eGFR <15 ml/min per 1.73 m2 and between 15 and 29 ml/min per 1.73 m2 had 1.4- and 1.8-fold higher risks of progression to ESRD compared to Caucasians |
| Grams et al., 2017 | US | To determine risk factors that explain the variability in prognosis and timing of adverse outcomes (e.g.: CVD, ESRD) for people with advanced CKD | 1,798 | Adults aged 21 to 74 and with CKD | eGFR<30ml/min/1.73m^2^ | Caucasian, African American | Prospective cohort study | Cox regression model, competing risk proportional hazard model | End-stage renal disease, cardiovascular disease (congestive heart failure, stroke, myocardial infarction, and peripheral artery disease), mortality | Age, sex, eGFR, proteinuria, diabetes mellitus, body mass index, ejection fraction, systolic blood pressure, history of CVD, and smoking history. | For all participants, the development of ESRD or CVD increased the risk for subsequent mortality, with no differences by patient race. Sub hazard ratios for first CVD, ESRD, and death events for African-Americans compared to Caucasians was 0.90 (0.71–1.14), 1.16 (0.93–1.43), and 1.69 (1.09–2.62), respectively. Prognosis and timing of adverse outcomes in chronic kidney disease varied by patient characteristics such as race and sex. |
| Go et al., 2018 | US | to identify predictors of fast progression during the first 2 years of follow-up in the presence or absence of diabetes mellitus | 36,195 | older adults (mean age 73) in the San Francisco and greater Bay Area | eGFR 30-59ml/min per 1.73 m^2^ | Caucasian, African-American, Native Americans, Asian/Pacific Islanders | Retrospective cohort study | Multivariable logistic regression | CKD progression | age, gender, myocardial infarction, heart failure, valvular heart disease, coronary revascularization (coronary artery bypass surgery, percutaneous coronary intervention), pacemaker placement, atrial fibrillation and/or flutter, ischemic stroke, transient ischemic attack, peripheral artery disease, cardiovascular risk factors (tobacco usage, diabetes mellitus, hypertension, dyslipidaemia), other comorbid conditions (cancer, chronic liver disease, chronic lung disease, dementia, depression, extracranial haemorrhage, thyroid disease), ambulatory systolic and diastolic blood pressure, heart rate and body mass index, documented proteinuria, ambulatory level of haemoglobin, LDL cholesterol, HDL cholesterol and serum potassium | There were no significant ethnic differences in CKD progression. Compared to Caucasians, adjusted OR (95% CI) for fast progression in adults with stage 3 CKD and no diabetes was 1.02 (0.90-1.16), 1.00 (0.88-1.15),1.57 (0.81-3.04) for African-Americans, Asian/Pacific Islanders, Native Americans, respectively. Compared to Caucasians, adjusted OR (95% CI) for fast progression in adults with stage 3 CKD and no diabetes was 1.00 (0.88-1.14), 1.02 (0.91-1.15),1.22 (0.67-2.20) for African-Americans, Asian/Pacific Islanders, Native Americans, respectively. |
| Hall et al., 2010 | US | Examine patient characteristics, incident ESRD, and mortality | 15353 | Adults (aged 20 years and over) with nondialysis-dependent CKD stages 3 to 5 receiving regular ambulatory care in the Community Health Network between 1996 and 2005 | CKD stage 3-5 | African-Americans, Hispanics, Asians/Pacific Islanders, non-Hispanic Caucasian | Prospective cohort study | Cox proportional hazards regression model | Time to ESRD, time to death | Patient age, sex, health insurance coverage (uninsured, Medicaid, Medicare, or commercial/other), primary spoken language (English, Spanish, Cantonese, or other), housing status (domiciled or homeless), occupational status (employed, unemployed, disabled or retired), annual income, diabetes, hypertension, congestive heart failure, cardiovascular disease (defined as coronary artery, cerebrovascular, or peripheral vascular disease), chronic obstructive lung disease, hepatitis B virus (HBV), hepatitis C virus (HCV), HIV or AIDS, depression, tobacco smoking, alcoholism, and drug abuse | In adjusted analyses, African-Americans [HR (95% confidence interval), 4.00 (2.99 to 5.35)], Hispanics [2.20 (1.46 to 3.30)], and Asians/Pacific Islanders [3.84 (2.73 to 5.40)] had higher risks of progression to ESRD than non-Hispanic Caucasians. Higher risk of progression to ESRD in non-Caucasians was not explained by lower relative mortality. No significant association between race and risk of mortality in fully adjusted models, except for Asians/Pacific islanders having lower risk of death than non-Hispanic Caucasians. Risk of death (adjusted HR (95% CI)) for African-Americans, Hispanics, Asians/Pacific Islanders compared to Caucasians was 0.99 (0.84, 1.16), 0.94 (0.74, 1.20), 0.76 (0.61-0.95), respectively. |
| Harding et al., 2017 | US | To review literature on ethnic disparities in CKD outcomes | review | African-Americans and Caucasians in the general population | All stages | Caucasians, African American | Literature review | n/a | Processes of care |  | African Americans are 10 times more likely to develop hypertension-related kidney failure and 3 times more likely to progress to kidney failure compared to Caucasians. Genetic factors such as increased salt sensitivity and reduced plasma renin in the face of upregulation of the intra-renal renin-angiotensin system (RAS) independent of systemic RAS may contribute to early onset and progression of CKD in AA. Socioeconomic and environmental factors including late referral contribute to racial disparities in CKD outcomes |
| Hebert et al., 1997 | US | To compare the effects of the usual versus low blood pressure goal on the GFR decline in African-American and Caucasians with moderate renal disease of diverse causes in the MDRD Study and compare the relationship between achieved blood pressure and GFR decline in these participants | 548 | Adults aged 18-70 years with evidence of chronic renal disease | eGFR: 25-55ml/min/1.73m^2^ and 13-24ml/min/1.73m^2^ | African-Americans, Caucasians | Randomized clinical trial | Correlational analyses; mixed effects regression models | eGFR decline | Urine protein excretion, a diagnosis of polycystic kidney disease, MAP, serum transferrin, serum total and HDL cholesterol, age, sex, body mass index, protein intake, and haemoglobin A1C | The mean (±SE) GFR decline over 3 years in the low blood pressure group was 11.8±7.3 mL/min slower than in the usual blood pressure group among African-Americans (P=.11) compared with 0.3±1.3 mL/min slower among Caucasians (P=.81) (P=.12 between African-Americans and Caucasians). In both African-Americans and Caucasians, higher baseline urine protein excretion was associated with a greater beneficial effect of the low mean arterial pressure (MAP) goal on GFR decline (P=.02 for both races). Combining both blood pressure groups and controlling for baseline characteristics, higher follow-up achieved MAP was associated with faster GFR decline in both African-Americans (P<.001) and Caucasians (P=.002), with a sevenfold stronger relationship in African-Americans (P<.001). |
| Horowitz et al., 2015 | US | CKD progression and blood pressure management in people with CKD and hypertension |  | People with CKD | All stages | Caucasian, Hispanic, African-American | Literature review | n/a | CKD progression and blood pressure management in people with CKD and hypertension |  | Control of blood pressure (BP) in Hispanic and African Americans with CKD is worse than it is Caucasians. There are disparities in the patterns of treatment and rates of progression of CKD in patients with HTN. The presence and severity of CKD increase treatment resistance |
| Hsu et al., 2003 | US | To assess whether ethnic differences in ESRD is due to differences in prevalence of chronic renal insufficiency or increased progression in African-American compared to Caucasians | 3894000 | Nationally representative National Health and Nutrition Examination Surveys (NHANES) and the United States Renal Data System (USRDS) | CKD stage 3-4; eGFR: 15-59 mL/min/1.73 m2 | Caucasian, African-American | Birth cohort analysis | Cox proportional hazards regression model | Progression to ESRD | Age, sex, diabetes | Similar prevalence of chronic renal insufficiency (CRI) for African-American and Caucasians. There was increased risk of developing ESRD in African-American compared to Caucasians (risk ratio (RR): 4.8, 95% CI: 2.9-8.4), which was modestly affected by adjustment for age, gender, and diabetes. African-American with CRI had higher systolic and diastolic blood pressure (p=0.02) and higher albuminuria (0.01) than Caucasians with CRI. No statistical differences in use of angiotensin converting enzyme inhibitors (24% versus 18%; P =0.19) or the level of glycaemic control among the African-American and Caucasian CRI subjects who also had diabetes (glycated haemoglobin, 8.5% versus 8.2%; P =0.46) |
| Hsu et al., 2005 | US | To quantify the risk of chronic kidney disease progression associated with APOE in a population-based study including Caucasian, African American, diabetic, and nondiabetic individuals. | 14520 | Adults aged 45 to 64 years from 4 US communities: Forsyth County, North Carolina; Jackson, Miss; suburbs of Minneapolis, Minn; and Washington County, Maryland | No detail | Caucasian, African-American | Prospective cohort study | Proportional hazards model | Chronic kidney disease progression (an increase in creatinine of at least 0.4 mg/dL (35 μmol/L) above baseline or a hospitalization (discharge or death) coded for chronic renal disease) | Sex, diabetes, hypertension | Risk of CKD progression for African Americans was 2.31 (95% CI, 2.04-2.61) times that of Caucasians, after adjustment for age and sex. Further inclusion of APOE in the model showed that it did not explain the excess risk of CKD progression in African Americans (APOE-adjusted RR, 2.36; 95% CI, 2.09-2.68). There were no differential effects of APOE variation by race. Race-stratified analyses demonstrated that effects of APOE alleles on CKD progression were slightly stronger in African Americans but not significantly different by racial group. In multivariate analyses, ε2 APOE alleles conferred risk (RR, 1.07; 95% CI, 0.93-1.24) and ε4 APOE alleles was protective (RR, 0.87; 95% CI, 0.77-0.98), independent of major CKD risk factors (likelihood ratio test, P =.03) including hypertension and diabetes. ε2 was not associated with hospitalizations or end-stage renal disease |
| Hull et al., 2011 | UK | Assess the effect of ethnicity on the prevalence and management of hypertension and associated chronic kidney (CKD) disease | 49203 | Adults registered with GP practices in east London Primary Care Trusts (Newham, Tower Hamlets, City & Hackney) | CKD stage 3a-5 | Afro-Caribbean, Caucasians, South Asians | Cross-sectional study | Logistic regression model | Prevalence hypertension, CKD, blood pressure control, hypertensive-drug prescribing | Age, sex, mean systolic blood pressure, mean | Among those with eGFR < 60ml/min/1.73m2, South Asians had significantly greater risk of severe CKD (stages 4-5, eGFR<30ml/min/1.73m compared to Caucasians), after adjusting for systolic blood pressure, total cholesterol, smoking status, ischaemic heart disease, (OR: 1.44 (95% CI: 1.00, 1.85)). Among those with eGFR <60ml/min/1.73m^2^, higher proportion of South Asians than Caucasians or Afro-Caribbean achieved target blood pressure (worst for Afro-Caribbean) and higher proportion of Afro-Caribbean than Caucasians or South Asians were prescribed calcium channel blockers, alpha and beta blockers. Prescribing pattern was similar for Caucasians and South Asians with eGFR< 60ml/min/1.73m^2^, except slightly lower proportion of South Asians were prescribed alpha blockers. |
| Hunsicker et al., 1997 | US | Investigate which baseline factors are individually predictive of CKD progression | 840 | Adults aged 18-70 years with evidence of chronic renal disease | eGFR: 25-55ml/min/1.73m^2^, 13-24ml/min/1.73m^2^ | African-American, non-African-American | Randomized prospective trial | Mixed-effects regression model | Renal function decline | Age, education, body mass index, left ventricular hypertrophy, protein urine, blood pressure, renal function, albumin, lipids (total cholesterol), haemoglobin | Among patients with GFRs ranging from 25 to 55 ml/min per 1.73 m^2^, African-American race (compared to nonAfrican-American) independently predicted faster GFR decline - mean change (SE) in eGFR slope was -1.50 (0.66)ml/min/1.73m^2^ (p=0.03). |
| Jawadi et al., 2018 | US | to assess the difference in the prevalence and progression of diabetic nephropathy, and the development of end-stage renal disease (ESRD) in people from three different ethnic groups with Type 2 diabetes (T2DM | review | Patients with Type 2 diabetes mellitus and diabetic nephropathy | No detail | Caucasian, South Asian, Afro-Caribbean | Systematic review and meta-analysis | random effects meta-analyses models | ESRD |  | the pooled incidence rate ratio for ESRD in African Caribbean compared with Caucasian participants was significantly higher (IRR: 2.74 (95% CI: 2.01,3.48). There was no significant difference in incidence rate of ESRD for South Asians compared to Caucasians (IRR: 0.88 (95% CI: -0.18,1.94) |
| Jolly et al., 2014 | US | Calculated proportions of patients with and without CKD recognition in an electronic health record based CKD registry and examined differences by demographics, clinical factors, and development of ESRD or mortality | 49663 | Individuals 18 years and older with high blood pressure, diabetes or a family history of kidney failure and CKD stage 3-4 (Kidney Early Evaluation Program participants ) | All stages: urinary albumin to creatinine ratio (ACR) of ≥30 mg/g among participants with eGFR ≥60 ml/min per 1.73m^2^ or eGFR<60ml/min/1.73m^2^ | Caucasian, African American, Other | Prospective cohort study | Logistic regression model | Processes of care/quality of care, ESRD, all-cause mortality | Age, sex, race, insurance, eGFR, diabetes, hypertension, coronary artery disease, heart failure , hyperlipidaemia and cerebrovascular disease. | African American race compared to Caucasian race (OR: 2.20, 95% CI: 1.95-2.50), diabetes, hypertension, and cerebrovascular disease were independently associated with significantly higher CKD recognition. There was no independent association of CKD recognition with ESRD or mortality |
| Jones-Burton et al., 2005 | US | Examine effects of smoking and ethnicity on anaemia management in CKD in patients | 1312 | Adult (aged 18+ years) CKD patients who started a weekly dose of epoetin alfa for 16 weeks | Various stages of CKD | African American, non-African American | Prospective cohort study | Multiple regression models | Response to epoetin alfa, eGFR decline, blood pressure | Age, gender, baseline weight, presence of diabetes mellitus, cumulative epoetin alfa dose, baseline Hb, baseline eGFR, baseline serum ferritin, and systolic and diastolic blood pressures | African-Americans experienced a significant decline in eGFR when compared to non-African-Americans (-1.11±0.37 vs 0.003±0.271, P=0.02). After adjusting for potential confounders, African-American non-smokers had a diminished response to anaemia management relative to African-American smokers and non-African-Americans, regardless of smoking status. African-American smokers with CKD exhibit a response to epoetin alfa comparable to patients of other races. |
| Koppiker et al., 1998 | UK | Assess ethnic differences in rate of progression to ESRF in individuals with diabetic nephropathy | 39 | Patients who attended the Nephrology Clinics at the Leicester General Hospital | No detail | Indo -Asians, Caucasians | Retrospective case review | Cox regression model | Rate of progression to ESRF (calculated as slope of log serum creatinine) | Age at referral, sex, mean HbA, systolic and diastolic blood pressures at referral, duration of diabetes to initial referral and referral serum creatinine | Rate of progression to ESRF was not significantly different between Indo-Asian and Caucasian patients (RR: 0.48 (0.07,3.28)) |
| Kovesdy et al., 2009 | US | To compare pre-dialysis mortality, incidence of end stage renal disease (ESRD), and slopes of estimated GFR (eGFR) in African-American versus Caucasian male patients with moderate and advanced non-dialysis-dependent CKD | 1243 | outpatients referred for evaluation and treatment of non dialysis dependent CKD at Salem Veterans Affairs Medical Center (VAMC) | CKD stages 1-5 | African-American, Caucasians | Prospective cohort study | Generalized linear mixed-effects ; Cox proportional hazards regression model | Mortality, ESRD, slopes of eGFR | Case mix (age, cardiovascular disease including coronary artery, peripheral vascular and cerebrovascular disease, systolic and diastolic BP, diabetes and smoking), biochemistries (eGFR, serum albumin and bicarbonate, blood cholesterol, haemoglobin, WBC, percent lymphocytes, and 24 h urine protein), and medication use (calcitriol, calcium containing phosphate binders, sevelamer hydrochloride, and statins) | Slopes of eGFR were similar in Afro-Carribbeans and Caucasians. Afro-Carribbeans had lower crude mortality and higher crude ESRD incidence (unadjusted ESRD hazard ratio, 95%CI: 1.64 [1.28 to 2.12], P < 0.001). The lower mortality in Afro-Carribbeans compared to Caucasians (HR (95% CI): 0.75 (0.59-0.95)) was attenuated by differences in case mix (fully adjusted HR (95%CI): 1.03(0.80-1.34)), especially a lower prevalence of cardiovascular disease, and the higher incidence of ESRD was explained by differences in case mix and baseline kidney function (fully adjusted HR (95%CI):1.06 (0.78,1.43)) |
| Lewis et al., 2015 | US | Assess the influence of race on cardiovascular (CV) outcomes and incidence of ESRD | 4038 | Patients with Type 2 diabetes, CKD and anaemia | eGFR: 20 to <60ml/min/1.73m^2^ | Aborigine, American Indian or Alaskan, Asian, African American, Hispanic or Latino, Japanese, Native Hawaiian or Pacific Islander, Caucasian, or other | Randomized, double-blind, placebo-controlled study | Mixed-effects regression model, Cox proportional hazards model | End-stage renal disease, mortality (cardiovascular/non-cardiovascular related mortality) | Age, sex, heart failure, log (urinary protein-creatinine ratio), serum C-reactive protein, electrocardiographic abnormality, serum albumin, coronary heart disease, arrhythmia, serum HbA1C, blood reticulocytes, serum urea nitrogen, insulin use, cerebrovascular disease, loop diuretic use, serum haemoglobin, smoking status, blood transfusion, heart rate (per 10 beats), peripheral artery disease, body mass index (per 10 kg/m2), blood white cell count, hyperuricemia/gout, gastrointestinal bleeding in past 5 years, systolic blood pressure (per 10 mm Hg), eGFR (per 10 mL/min per 1.73 m2), lung disease, diabetes complications, duration of diabetes, and treatment randomization (darbepoetin alfa vs placebo) | During a mean follow-up of 2.4 years with comparable access to care, African-Americans and Hispanics had a greater risk of ESRD but a significant lower risk of myocardial infarction and coronary revascularization than Caucasians. After adjusting for confounders (body mass index, insulin use, eGFR, serum urea nitrogen, log (urinary protein-creatinine ratio), serum albumin, prior stroke, prior peripheral arterial disease, prior heart failure, cardiac arrhythmia, serum haemoglobin, log (serum ferritin), serum C-reactive protein, history of acute kidney injury, systolic blood pressure, and diastolic blood pressure) , African-Americans remained at significantly greater risk of ESRD than Caucasians (HR: 1.53, 95% CI 1.26-1.85, P < .001), whereas this ESRD risk did not persist among Hispanics. Despite similar access to care and lower CV event rates, the risk of ESRD was higher among African-Americans and Hispanics than Caucasians. Mortality was numerically lower for African-American and Hispanic patients both before and after ESRD (Table III), but this was not statistically significant (p for interaction = .19). |
| Lucas et al., 2008 | US | To examine the racial differences in the incidence and progression of HIV-related chronic kidney disease (CKD) that underlie African American–Caucasian disparities in HIV-related end-stage renal disease (ESRD) | 4259 | HIV-infected adults who received care from John Hopkins clinic | eGFR>15ml/min/1.73m^2^ | African American, Caucasian | Prospective cohort study | Cox proportional hazards regression model | Progression to ESRD, GFR slope | Age, baseline eGFR, albumin at onset of CKD, haemoglobin, proteinuria, use of ACE-I or ARB | Among those who had CKD, the African American subjects developed ESRD markedly faster than did the Caucasian subjects (HR, 17.7 [95% CI, 2.5–127.0]), and, correspondingly, their GFR decline after diagnosis of CKD was 6-fold more rapid (P <.001). Rate of progression was faster for African Americans with CKD than Caucasians with CKD, irrespective of the presence of HIV-associated nephropathy. |
| Mathur et al., 2018 | UK | To determine ethnic differences in the progression of chronic kidney disease (CKD) and risk of end-stage renal failure (ESRF) and death in adults with type 2 diabetes mellitus (T2DM), and to identify predictors of rapid renal decline. | 6274 | General practice-registered adults aged 25–85 years with established T2DM and CKD at baseline. | CKD stage 3 or below | Caucasian, South Asian, Afro-Caribbean | Prospective cohort study with nested case-control study | multilevel linear regression (rate of decline); logistic regression (odds of decline); Cox proportional hazards regression | Progression to ESRD, GFR slope | age, gender, Index of Multiple Deprivation score, systolic blood pressure (SBP), glycated haemoglobin (HbA1c), proteinuria, smoking status, BMI, diagnosed cardiovascular disease (ischaemic heart disease, heart failure, stroke, peripheral vascular disease) and any prescription of ACE inhibitors and angiotensin receptor blockers (ARBs). | Age-sex adjusted rate of eGFR decline was fastest in South Asian (coeff (95% CI): -0.77(-0.81, -0.74) than Caucasians (-0.64 (-0.68,-0.60) and Afro-Caribbeans (0.55(-0.61,-0.48). After adjusting for confounders, African-Caribbean and South Asians had higher odds of faster progression. Adjusted OR for African-Caribbean compared to Caucasian: 2.53 (95% CI: 1.63-3.92). The risk of ESRD was more than doubled in African-Americans compared to Caucasians in a fully adjusted analysis which also took account of the competing risk of death (HR 2.23, 95% CI 1.25 to 2.98). Adjusted risk for death, compared to Caucasians, was HR (95% CI): 0.71 (0.56 to 0.90) and 0.77 (0.57 to1.05) for South Asian and Afro-Caribbeans, respectively. |
| Menon et al., 2008 | US | Examine renal outcomes in non-diabetic participants with CKD stage 2-4 | 1666 | Nondiabetic participants from the Modification of Diet in Renal Disease (MDRD) study | CKD stage 2-4 | African-Americans, non-African-Americans | Retrospective cohort study | Competing risk models | Kidney failure, mortality | Subgroup analysis by GFR, proteinuria, kidney disease aetiology, sex | Rates for kidney failure and death (both before and subsequent to kidney failure) were similar for men and women, and for African-Americans versus non-African-Americans, and kidney failure was the more likely outcome in each of these groups. Rate (per 1000 person-years, 95% CI) for kidney failure was 85 (79-90) and 80 (65-96) for Caucasians and African-Americans, respectively. Rate (per 1000 person-years, 95% CI) for death was 23 (20-25) and 25 (18-32) for Caucasians and African-Americans, respectively. |
| Norris et al., 2008 | US | Commentary on studies exploring ethnic differences in CKD progression and mortality |  | African-Americans and Caucasians in the general population | All stages | non-Hispanic Caucasian, African American, Asian, American Indian/Alaska Native, and Hispanic | Commentary | n/a | CKD progression to end-stage renal disease, cardiovascular and non-cardiovascular-related mortality |  | Biological, environmental, sociocultural, and healthcare system factors contribute to ethnic disparities in CKD outcomes |
| Pallayova et al., 2015 | UK | Examine potential ethnicity-related differences in progression of chronic kidney disease (CKD) between South Asian and Caucasian European diabetic adults with CKD stage 3 over a 5-year period. | 1173 | Diabetic adults who had attended diabetes and diabetes-renal outpatient clinics | eGFR≥ 30ml/min/1.73m^2^ and <60ml/min/1.73m^2^ | European Caucasian, South Asian | Prospective cohort study | Correlational analyses and regression models | eGFR decline | Age, sex, baseline creatinine, baseline eGFR, baseline albumin to creatinine ratio, baseline HbA1c, baseline total cholesterol | The 5-year follow-up eGFR and the decline in eGFR did not differ between the two groups. Thirty-five (12.4%) South Asians and 82 (9.2%) Caucasian Europeans progressed to stages 4–5 CKD (P = 0.112) |
| Parsa et al., 2013 | US | Examine the effects of variants in the gene encoding apolipoprotein L1 (APOL1) on the progression of chronic kidney disease in Caucasian and African-American people | 2955 | Patients from the African American Study of Kidney Disease and Hypertension (AASK) and Chronic Renal Insufficiency Cohort (CRIC) | eGFR: 20-70ml/min/1.73m^2^ | African-Americans, Caucasians | Prospective cohort study | Mixed-effects and Cox proportional-hazards model | Rate of eGFR decline, a composite of ESRD and 50% reduction in baseline eGFR, mortality | Age, sex, clinical site, and baseline eGFR, education level, treatment by a nephrologist, and use of either an ACE inhibitor or angiotensin-receptor blocker [as a proxy for treatment access, systolic blood pressure, body-mass index, glycated haemoglobin level, and smoking status, total 24-hour urinary protein excretion | African-American patients in the APOL1 high-risk group had a more rapid decline in eGFR and a higher risk of the composite renal outcome than did Caucasian patients and African-American patients in the low APOL1 risk group, among those with diabetes (-0.79 (-1.41 to -0.17)ml/min/1.73m^2^ )for African-Americans compared to Caucasians, p=0.01) and those without diabetes (-0.81 (-1.26 to -0.35) ml/min/1.73m2 for African-Americans compared to Caucasians). Death rates for African-American patients were similar to those for Caucasian patients. |
| Peralta et al., 2006 | US | Examine the associations between Hispanic ethnicity and risks for ESRD, cardiovascular events, and death in patients with CKD. | 39550 | Patients with stages 3 to 4 CKD from Kaiser Permanente of Northern California | CKD stages 3 to 4 | Hispanics, non-Hispanic Caucasians | Prospective cohort study | Cox regression models | ESRD, cardiovascular events, mortality | Age, gender, income, education, and preferred language, hypertension and medical history, diabetes and use of insulin, baseline eGFR and time-updated proteinuria, time-varying medication used | After adjustment for sociodemographic characteristics, Hispanic ethnicity was associated with an increased risk for ESRD (hazard ratio [HR] 1.93; 95% confidence interval [CI] 1.72 to 2.17) when compared with non-Hispanic Caucasian patients, which was attenuated after controlling for diabetes and insulin use (HR 1.50; 95% CI 1.33 to 1.69). After further adjustment for potential confounders, Hispanic ethnicity remained independently associated with an increased risk for ESRD (HR 1.33; 95% CI 1.17 to 1.52) as well as a lower risk for cardiovascular events (HR 0.82; 95% CI 0.76 to 0.88) and death (HR 0.72; 95% CI 0.66 to 0.79). |
| Salifu et al., 2009 | US | to assess if increased progression of diabetic kidney disease in African-Americans compared to Caucasians is due to poorer glycaemic control in African-Americans | 183 | Patients with diabetic kidney disease | CKD stage 1 to 4 | African-Americans, Caucasians | Prospective cohort study | Linear regression models | Decline in eGFR | Age, sex, haemoglobin, creatinine | There were no significant differences in eGFR at any time point between African-Americans and Caucasians in any stage of CKD. Under equivalent glycaemic control, there were no ethnic differences in rate of progression of diabetic kidney disease |
| Samuel et al., 2014 | Canada | To examine whether presence and severity of albuminuria contributes to progression of chronic kidney disease to kidney failure among First Nations and non-First Nation people | 1816824 | Adult (18+ years) residents of Alberta | eGFR <60ml/min/1.73m^2^; 15-29.9ml/min/1.73m2, 30-44.9ml/min/1.73m2, 45-59.9ml/min/1.73m2 | First Nation, non-First nation | Prospective cohort study | Cox proportional hazards model | Progression to kidney failure (defined as chronic dialysis, renal transplantation, or sustained doubling of serum creatinine) | Age, sex, diabetes, hypertension, location of residence, income quintile, specialist care, diabetes, hypertension, cerebrovascular disease, peripheral vascular disease, congestive heart failure, cancer, COPD, dementia, Metastatic solid tumour, myocardial infarction, liver disease, Paralysis, peptic ulcer disease, rheumatic disease | Rates of progression to kidney failure were consistently 2- to 3-fold higher among First Nations people than among non–First Nations people, across all levels of albuminuria and estimated GFRs. Compared with non–First Nations people, First Nations people with an estimated GFR of 15.0–29.9 mL/min per 1.73 m2 (HR (95% CI): 18.67 (10.77-32.36) vs 6.33 (5.41 -7.40) for First Nation vs non First Nation, respectively) had the highest risk of progression to kidney failure, with similar hazard ratios for those with normal and heavy albuminuria |
| Van den Beukel et al., 2013 | Norway | Compare progression from CKD to ESRD for patients from different ethnic groups receiving pre-dialysis care in a universal health care system | 995 | Patients with CKD who started pre-dialysis care in The Netherlands (1999–2011) | CKD stages 4-5: eGFR<20–30 ml/min per 1.73 m^2^ | Caucasians (Europeans, Turkish, Moroccan), Afro-Caribbean (sub-Saharan African origin, including Surinamese creoles) | Prospective cohort study | Cox proportional hazards; linear mixed models | Time to initiation of renal replacement therapy, renal function decline (2 years follow up) | Age, sex, BMI, blood pressure, smoking, diabetes, CVD, ACE/ARB medication, eGFR and proteinuria at baseline | No difference in time to the start of RRT within the first 15 months of pre- dialysis care between Afro-Caribbean and Caucasian incident patients starting pre-dialysis care. Afro-Caribbean patients initiated pre-dialysis care with a higher eGFR than Caucasians. From 15 months onward, Afro-Caribbeans had a 1.93-fold higher hazard (95% CI: 1.02, 3.68) of starting RRT compared with Caucasians. Adjustment for differences in demographic characteristics, comorbid conditions and lifestyle, prescribed medication, proteinuria, eGFR, and laboratory measurements at baseline increased this HR (95% CI) to 3.12 (1.56 , 6.23). Renal function decline was 0.18 (95% CI, 0.05 to 0.32) ml/min per 1.73 m2 per month faster in Afro-Caribbean than in Caucasian patients and remained faster after adjustment |
| Yang et al., 2014 | US | Assess association of risk factors with CKD progression indicated by ESRD, eGFR decline or death | 3939 | Adults aged 21 to 74 and with CKD (Chronic Renal Insufficiency Cohort (CRIC) study) | Mild to moderate CKD | Caucasian, African-American, other | Prospective cohort study | [Multivariable Cox proportional hazards models](https://www.ncbi.nlm.nih.gov/pmc/articles/PMC3946885/#R14) | eGFR decline, ESRD, mortality | Age, race, sex, diabetes, baseline estimated glomerular filtration rate (eGFR), proteinuria, ankle brachial index, uric acid, history of CVD, BMI, hypertension, haemoglobin, education and smoking status. | Older age, diabetes, lower eGFR, and high proteinuria levels were associated with increased risk of death, female sex associated with lower risk of death and no significant association between race and risk of death (HR (95% CI): 1.09 (0.89,1.33) for African-Americans compared to Caucasians). Older age, female sex were associated with lower rates of ESRD. The HRs for ESRD among African-American and those of other races/ethnicity compared to Caucasians were 1.55 (95% CI, 1.29–1.86) and 1.52 (95% CI, 1.17–1.98), respectively. The HRs for eGFR halving among African-American and those of other races/ethnicity compared to Caucasians were 1.43 (95% CI, 1.22–1.68) and 1.44 (95% CI, 1.14–1.82), respectively |

**Mortality**

| **Author & year** | **Country** | **Study Aims** | **N** | **Study population** | **CKD stage/eGFR** | **Ethnic groups** | **Study design** | **Analysis** | **Outcomes** | **Adjusted confounders** | **Conclusions** |
| --- | --- | --- | --- | --- | --- | --- | --- | --- | --- | --- | --- |
| Agarwal et al., 2008 | US | To evaluate the competing risk of ESRD versus mortality and to evaluate the risk factors associated with these two outcomes in a CKD population | 220 | Patients at a Veterans Administration Medical Centre | eGFR <60ml/min/1.73m^2^ or urine protein/creatinine ration >0.22g/g | Caucasian, African American | Prospective cohort study | Competing risk Cox regression models | End-stage renal disease | Age, proteinuria, eGFR, systolic blood pressure, and coronary artery disease | African-American had higher risk of ESRD [adjusted HR (95% CI): 2.75 (1.09-6.92)] but similar risk of all-cause mortality [adjusted HR (95% CI): 1.15 (0.60-2.17)] |
| Ali et al., 2013 | UK | Compare the rate of progression of diabetic CKD in different ethnic groups | 329 | All new adult patients attending a tertiary renal unit in east London with diabetic chronic kidney disease between 2000 and 2007 and followed up till 2009 were included. Patients presenting with acute end-stage kidney failure were excluded. | No detail | South Asian, Caucasian, African-American | Prospective cohort study | linear regression; Cox regression model | Annual decline in eGFR, progression to ESRD, and mortality in different ethnic groups | Baseline eGFR, systolic and diastolic blood pressure, proteinuria, ACE or angiotensin receptor blockers use, and glycated haemoglobin, ischemic heart disease, CVD, diabetic retinopathy | Annual decline in eGFR, numbers progressing to ESRD and total mortality not significant different across the 3 groups. Compared to Caucasians, adjusted mean annual decline was (coefficient from adjusted model:) -1.67 (-4.97 to 1.64) and -0.51 (-3.25 to 2.23) for African-Americans and South Asians, respectively. Risk of death for Afro-Caribbeans and South Asians compared to Caucasians was 1.01 (0.63-2.57) and 1.17 (0.68-2.32), respectively. |
| Alves et al., 2010 | US | Assess prevalence, incidence, rapid eGFR decline (≥50% decrease in baseline value), ESRD, and mortality among HIV-associated CKD | 2468 | HIV-infected adults from a non-profit clinic in Tennessee | eGFR <60ml/min/1.73m^2^ | Non-African-Americans and African-Americans | Retrospective cohort study | Mixed effects, competing risks, Poisson and Cox regression | eGFR decline, ESRD, mortality | Sex, race, baseline age, level of kidney function, anaemia, cardiovascular disease, absolute CD4 lymphocyte count (CD4), HIV-1 RNA viral load, history of angiotensin-converting enzyme inhibitor or angiotensin receptor blocker, HAART, opportunistic infection, hypertension (HTN), chronic hepatitis C, diabetes mellitus, and HIV risk group: men-having-sex-with-men, heterosexual contact, intravenous drug use, other, or unknown | Risk for rapid eGFR decline was similar for both groups. African Americans (AA) had significantly higher risk of ESRD and were more likely to die than non-African-Americans, although not statistically significant. For eGFR decline outcome, adjusted HR for AA compared to non-AA was 2.5 (0.9, 6.9). For death outcome, adjusted HR for AA compared to non-AA was 1.9(0.6, 6.5) |
| Babayev et al., 2013 | US | Explore whether race and body mass index (BMI) influence end-stage renal disease (ESRD) and mortality rates in participants with CKD stages 3-4 | 14,631 | Individuals 18 years and older with high blood pressure, diabetes or a family history of kidney failure and CKD stage 3-4 (Kidney Early Evaluation Program participants) | CKD stage 3-4; eGFR: 15-59 mL/min/1.73 m^2^ | Caucasian, African-American | Prospective cohort study | Survival analyses model | End-stage renal disease, mortality | Age, sex, hypertension, diabetes, baseline eGFR, albuminuria | African-American race was associated with higher incidence of ESRD, but not death. In multivariable models, African American race increased the risk of ESRD (HR, 1.66; 95% CI, 1.26-2.07), but not death (HR, 0.89; 95% CI, 0.76-1.03). Male sex, hypertension, diabetes, lower baseline eGFR, and albuminuria were predictive of higher rates of ESRD. Age, male sex, diabetes, lower baseline eGFR and albuminuria were predictive of overall mortality. No significant interaction between race and BMI in progression to ESRD or death |
| Barbour et al., 2010 | Canada | Compare CKD progression across different ethnic groups | 3444 | CKD adult patients referred to nephrologist | eGFR < 60 mL/min/1.73 m^2^ alone or, if eGFR > 60 mL/min/1.73 m2, evidence of kidney damage from urinalysis or based on biopsy or ultrasound results | Caucasian, East Asian, South Asian | Prospective cohort study | Survival analyses (Cox proportional hazard analysis) | Time to death, renal replacement therapy, rate of decline in eGFR | Blood pressure, proteinuria, age, sex, diabetes, CVD, medications (ACEI, ARB, statins or vitamin D analogues) | Caucasians higher risk of death than South Asian or East Asian (HR (95% CI): 0.69 (0.55-0.88) and 0.80 (0.63-1.02). East Asian and South Asian have higher risk of progressing to RRT and lower risk of death before RRT (HR: 0.72 and 0.52, p<0.05) compared to Caucasians) after adjusting for usual risk factors (age, sex, diabetes, cardiovascular disease (CVD),blood pressure, baseline eGFR levels and use of ACEI/ARB/vitamin D/statins). However, after adjusting for laboratory abnormalities (renal comorbidities), differences for progression were not significant, but survival advantage for Asians remained significant. |
| Cardarelli et al., 2008 | US | Assess whether CKD affects rate of death and major adverse cardiovascular events (myocardial infarction, revascularization, and death) differently in African-American and Caucasian patients a year after percutaneous interventions | 916 | Patients referred for percutaneous coronary interventions (PCIs) in the Emory Healthcare System between 2001-2004 | eGFR<60ml/min/1.73m^2^ | Caucasian, African-American | Prospective cohort study | Cox proportional hazards model | mortality, CVD | age, sex, race, diabetes, eGFR, hypertension, left ventricular ejection fraction, prior coronary artery bypass grafting, and prior percutaneous coronary intervention | African-American and Caucasian patients with CKD have similar mortality rate (HR (95% CI) for Caucasians compared to African-Americans: 1.88 (0.98-3.62)) and major adverse cardiovascular events (HR (95% CI) for Caucasians compared to African-Americans: 0.93 (0.62-1.40)) at 1 year post percutaneous coronary intervention |
| Choi et al., 2009 | US | Compare risk of ESRD and death by level of eGFR at baseline | 2,015,891 | US veterans registered within the Department of Veterans Affairs health system | eGFR levels, 45-60, 3-44, 14-29, <30 | Caucasian, African-American | Prospective cohort study | Cox proportional hazard model | ESRD, mortality | Age, sex, baseline comorbidities, and socioeconomic status, delivery of healthcare (health centre), eGFR level | Risk of ESRD was higher for African-American compared to Caucasians at all levels of eGFR (including those <60ml/min/1.73m2). The adjusted hazard ratios (HR) for end-stage renal disease associated with African-American versus Caucasian for patients with an eGFR 45-59, 30-44, 15-29, and <15 mL/min/1.73m2, respectively were 3.08 (95% CI, 2.74-3.46), 2.47 (95% CI, 2.26-2.70), 1.86 (95% CI, 1.75-1.98), and 1.23 (95% CI, 1.12- 1.34). Age standardized rate of death was higher for African-American than Caucasians - association of African-American race with mortality was strongest at 45 ml/min/1.73m2 and was progressively attenuated at lower levels of eGFR. The highest risk of mortality associated with African-American ethnicity was also observed among those with an eGFR 45-59 mL/min/1.73m2 (HR 1.32, 95% CI, 1.27-1.36). |
| Conley et al., 2012 | Canada | Explore the association between proteinuria, estimated glomerular filtration rate (eGFR), and risk of mortality and kidney failure in Caucasian, Chinese, and South Asian populations | 491,729 | adult (aged 20^+^ years) who had at least one outpatient serum creatinine measurement in 2005 | eGFR<60ml/min/1.73m^2^ | Caucasian, Chinese, or South Asian ethnicity | Prospective cohort study | Poisson regression models | Prevalence of proteinuria, all-cause mortality | age, sex, diabetes, hypertension, socioeconomic status, income, history of cancer, cerebrovascular disease, congestive heart failure, chronic obstructive pulmonary disease, dementia, HIV/AIDS, metastatic solid tumour, myocardial infarction, liver disease, paralysis, peptic ulcer disease, peripheral vascular disease, rheumatic disease | For participants with eGFR <60 mL/min/1.73 m2, the prevalence of heavy proteinuria was higher in Chinese and South Asians compared with Caucasians. Compared with Caucasians, adjusted rates of death were significantly lower for Chinese and South Asian populations (rate ratios (RR), 0.67 [95% CI, 0.56-0.80] and 0.73 [95% CI, 0.59-0.88], respectively); |
| Derose et al., 2013 | US | To examine racial differences in projected kidney failure (based on linear decline in eGFR rate) and mortality among those with projected kidney failure | 1,119,816 | Adult members of Kaiser Permanente Southern California from 2003–2009 with >2 serum creatinine tests and >180 days between tests | eGFR<60, 29-15, 30-44,45-59 | Hispanic, African-American, Asian (including Pacific Islanders), Caucasian | Retrospective cohort study | Linear regression (eGFR decline); Competing risk proportional hazard regression (mortality) | ESRD/eGFR decline, mortality | Age, sex, baseline eGFR | Projected kidney failure during CKD stage 3 and 4 was higher amongst African-Americans, Hispanics, and Asians compared to Caucasians. The ORs for projected kidney failure vs. Caucasians during CKD stages 3 and 4 were 1.54 (95% CI, 1.46– 1.62) in African-Americans, 1.49 (95% CI, 1.42–1.56) in Hispanics, and 1.41 (95% CI, 1.32–1.51) in Asians. Mortality among those with projected kidney failure was higher for Caucasians compared to African-Americans, Hispanics, Asians. Among those with projected kidney failure, the HRs of death vs. whites during CKD stages 3 and 4 were 0.82 (95% CI, 0.77–0.88) in African-Americans, 0.67 (95% CI, 0.63–0.72) in Hispanics, and 0.58 (95% CI, 0.52–0.65) in Asians. |
| Fedewa et al., 2014 | US | Examine survival among individuals with CKD stage 3 or 4, by income and race | 2761 | non-institutionalised adults (aged 45+ years) (the Reasons for Geographic and Racial Differences in Stroke (REGARDS) study.) | CKD stage 3-4: eGFR: 15-59.9ml/min/1.73m^2^ | African-American, Caucasian | prospective cohort study | Cox proportional hazards model | mortality | age, gender, education, insurance, CKD stage, comorbidity and county-level poverty | Low income increased hazard of death for both African-Americans (HR: 1.53, 95% CI: 1.18-1.99) and Caucasians (HR: 1.38, 95% CI: 1.10-1.74). No significant interaction effect of income and race on hazard of death. African-Americans had higher adjusted hazard of mortality (HR=1.30, 95% CI:1.02-1.65) compared to Caucasians |
| Fischer et al., 2016 | US | Assess association between race, CKD progression, incident ESRD, mortality | 3785 | Adults with CKD | eGFR <60ml/min/1.73m^2^, <45 | Caucasian, African-American, Hispanic | Prospective cohort study | Cox proportional hazard analysis | CKD progression, ESRD | Age, sex, education, smoking, health insurance, received nephrology care at baseline, diabetes, systolic blood pressure, eGFR, 24-hour urine protein, body mass index, haemoglobin, ACEi/ARB use | Hispanics had significantly higher rates of CKD progression, incident ESRD, and mean annual decline in eGFR than did Caucasians (rate per 100 person years: 1.41 (p<0.001) but not African-Americans. . In adjusted analyses, the risk of CKD progression did not differ between Hispanics and Caucasians or African-Americans. However, among nondiabetic participants, compared with NHB, Hispanics had a lower risk of CKD progression (HR: 0.61; 95% confidence interval, 0.39 to 0.95) and incident ESRD (HR: 0.50; 95% confidence interval, 0.30 to 0.84). African-American patients with eGFR <15 ml/min per 1.73 m2 and between 15 and 29 ml/min per 1.73 m2 had 1.4- and 1.8-fold higher risks of progression to ESRD compared to Caucasians. Hispanics had similar risk of all-cause mortality compared to Caucasians (adjusted HR (95% CI): 0.89 (0.59-1.35) |
| Grams et al., 2017 | US | To determine risk factors that explain the variability in prognosis and timing of adverse outcomes (e.g.: CVD, ESRD) for people with advanced CKD | 1,798 | Adults aged 21 to 74 and with CKD | eGFR<30ml/min/1.73m^2^ | Caucasian, African American | Prospective cohort study | Cox regression model, competing risk proportional hazard model | End-stage renal disease, cardiovascular disease (congestive heart failure, stroke, myocardial infarction, and peripheral artery disease), mortality | Age, sex, eGFR, proteinuria, diabetes mellitus, body mass index, ejection fraction, systolic blood pressure, history of CVD, and smoking history. | For all participants, the development of ESRD or CVD increased the risk for subsequent mortality, with no differences by patient race. Subhazard ratios for first CVD, ESRD, and death events for African-Americans compared to Caucasians was 0.90 (0.71–1.14), 1.16 (0.93–1.43), and 1.69 (1.09–2.62), respectively. Prognosis and timing of adverse outcomes in chronic kidney disease varied by patient characteristics such as race and sex. |
| Hall et al., 2010 | US | Examine patient characteristics, incident ESRD, and mortality | 15353 | Adults (aged 20 years and over) with nondialysis-dependent CKD stages 3 to 5 receiving regular ambulatory care in the Community Health Network between 1996 and 2005 | CKD stage 3-5 | African-Americans, Hispanics, Asians/Pacific Islanders, non-Hispanic Caucasian | Prospective cohort study | Cox proportional hazards regression model | Time to ESRD, time to death | Patient age, sex, health insurance coverage (uninsured, Medicaid, Medicare, or commercial/other), primary spoken language (English, Spanish, Cantonese, or other), housing status (domiciled or homeless), occupational status (employed, unemployed, disabled or retired), annual income, diabetes, hypertension, congestive heart failure, cardiovascular disease (defined as coronary artery, cerebrovascular, or peripheral vascular disease), chronic obstructive lung disease, hepatitis B virus (HBV), hepatitis C virus (HCV), HIV or AIDS, depression, tobacco smoking, alcoholism, and drug abuse | In adjusted analyses, African-Americans [HR (95% confidence interval), 4.00 (2.99 to 5.35)], Hispanics [2.20 (1.46 to 3.30)], and Asians/Pacific Islanders [3.84 (2.73 to 5.40)] had higher risks of progression to ESRD than non-Hispanic Caucasians. Higher risk of progression to ESRD in non-Caucasians was not explained by lower relative mortality. No significant association between race and risk of mortality in fully adjusted models, except for Asians/Pacific islanders having lower risk of death than non-Hispanic Caucasians. Risk of death (adjusted HR (95% CI) for African-Americans, Hispanics, Asians/Pacific Islanders compared to Caucasians was 0.99 (0.84, 1.16), 0.94 (0.74, 1.20), 0.76 (0.61-0.95), respectively. |
| Hayes et al., 2012 | US | Assess if hypokalaemia and hyperkalaemia are associated with pre-dialysis mortality in CKD patients, and how this association differs by race | 1227 | patients referred for evaluation and treatment to the Nephrology Department at Salem Veteran Affairs Medical Center of non-dialysis-dependent CKD | CKD stages 1-5 | African-Americans, Caucasian | prospective cohort study | linear mixed-effects regression models; Cox regression models | pre-dialysis mortality | age, Charlson comorbidity Index, diabetes mellitus (DM), cardiovascular disease, BMI, smoking status, eGFR, serum albumin, bicarbonate, calcium, phosphorus, blood haemoglobin, 24-hour urine protein, and the use of ACEI/ARB, diuretics, potassium supplements, NSAIDs and beta-blockers | Both hypo- and hyperkalaemia were associated with mortality in Caucasian patients. Hyperkalaemia was not associated with mortality in African-Americans; hypokalaemia was associated with mortality in African-Americans. Hypokalaemia was a stronger predictor of mortality for African-Americans compared to Caucasians. Hypokalaemia was associated with loss of kidney function independent of race. The discrepant risk of mortality associated with a serum potassium >5.5 mEq/l in whites and blacks persisted when including only 258 white patients (81 of whom had a serum potassium >5.5 mEq/l) matched to black patients by potassium levels: multivariable adjusted hazard ratio (95% CI) in whites with potassium >5.5 vs. 3.8–5.5 mEq/l: 2.01 (0.98–4.13), p = 0.057. |
| Hutchison et al., 2014 | UK | To determine whether elevated serum polyclonal free light chain (FLC) levels predict mortality in a population of individuals with chronic kidney disease (CKD). | 848 | adult patients of kidney disease clinics at University Hospitals Birmingham | CKD stages 1-5 | Caucasian, South Asian, African Caribbean, other | prospective cohort study | Cox proportional hazards model | 1 year mortality | age, sex, ethnicity, CVD comorbidity (ischemic heart disease, cardiac failure, cerebrovascular disease, and peripheral vascular disease), BP, and the use of angiotensin-converting enzyme inhibitors or angiotensin receptor blockers, and serum laboratory variables (albumin, phosphate, calcium, creatinine, cystatin C, C reactive protein, eGFR, and serum FLC). | High combined FLC levels were an independent risk factor for death (hazard ratio [HR], 2.71; 95% CI, 1.98-3.70; P<.001). South Asian ethnicity (compared to Caucasian ethnicity) was associated with lower risk of mortality (HR, 0.33; 95% CI, 0.14-0.64; P¼.02) |
| Jolly et al., 2011 | US | explore racial/ethnic differences in survival | 19,205 | Individuals 18 years and older with high blood pressure, diabetes or a family history of kidney failure and CKD stage 3-4 (Kidney Early Evaluation Program participants ) | all stages: urinary albumin to creatinine ratio (ACR) of ≥30 mg/g among participants with eGFR ≥60 ml/min per 1.73m^2^ or eGFR<60ml/min/1.73m^2^ | non-Hispanic Caucasian, African American, Asian, American Indian/Alaska Native, and Hispanic | Retrospective cohort study | Cox proportional hazards regression model | all-cause mortality | age, sex, obesity, diabetes, hypertension, albuminuria, baseline eGFR, heart attack, stroke, smoking, family history, education, health insurance, geographic region, and year screened | African Americans had a similar risk of death compared with Caucasians (adjusted Hazard Ratio (AHR) 1.07, 95% CI 0.90 to 1.27). Hispanics (AHR 0.66, 95% CI 0.50 to 0.94) and Asians (AHR 0.63, 95% CI 0.41 to 0.97) had a lower mortality risk compared with Caucasians. In contrast, American Indians/Alaska Natives had a higher risk of death compared with Caucasians (AHR 1.41, 95% CI 1.08 to 1.84) |
| Jolly et al., 2014 | US | Calculated proportions of patients with and without CKD recognition in an electronic health record-based CKD registry and examined differences by demographics, clinical factors, and development of ESRD or mortality | 49663 | Individuals 18 years and older with high blood pressure, diabetes or a family history of kidney failure and CKD stage 3-4 (Kidney Early Evaluation Program participants ) | All stages: urinary albumin to creatinine ratio (ACR) of ≥30 mg/g among participants with eGFR ≥60 ml/min per 1.73m^2^ or eGFR<60ml/min/1.73m^2^ | Caucasian, African American, Other | Prospective cohort study | Logistic regression model | Processes of care/quality of care, ESRD, all-cause mortality | Age, sex, race, insurance, eGFR, diabetes, hypertension, coronary artery disease, heart failure, hyperlipidaemia and cerebrovascular disease. | African American race compared to Caucasian race (OR: 2.20, 95% CI: 1.95-2.50), diabetes, hypertension, and cerebrovascular disease were independently associated with significantly higher CKD recognition. There was no independent association of CKD recognition with ESRD or mortality |
| Kovesdy et al., 2009 | US | To compare pre-dialysis mortality, incidence of end stage renal disease (ESRD), and slopes of estimated GFR (eGFR) in African-American versus Caucasian male patients with moderate and advanced non-dialysis-dependent CKD | 1243 | outpatients referred for evaluation and treatment of non-dialysis dependent CKD at Salem Veterans Affairs Medical Center (VAMC) | CKD stages 1-5 | African-American, Caucasians | Prospective cohort study | Generalized linear mixed-effects ; Cox proportional hazards regression model | Mortality, ESRD, slopes of eGFR | Case mix (age, cardiovascular disease including coronary artery, peripheral vascular and cerebrovascular disease, systolic and diastolic BP, diabetes and smoking), biochemistries (eGFR, serum albumin and bicarbonate, blood cholesterol, haemoglobin, WBC, percent lymphocytes, and 24 h urine protein), and medication use (calcitriol, calcium containing phosphate binders, sevelamer hydrochloride, and statins) | Slopes of eGFR were similar in Afro-Carribbeans and Caucasians. Afro-Carribbeans had lower crude mortality and higher crude ESRD incidence (unadjusted ESRD hazard ratio, 95%CI: 1.64 [1.28 to 2.12], P < 0.001). The lower mortality in Afro-Carribbeans compared to Caucasians (HR (95% CI): 0.75 (0.59-0.95)) was attenuated by differences in case mix (fully adjusted HR (95%CI): 1.03(0.80-1.34)), especially a lower prevalence of cardiovascular disease, and the higher incidence of ESRD was explained by differences in case mix and baseline kidney function (fully adjusted HR (95%CI):1.06 (0.78,1.43)) |
| Kovesdy et al., 2013 | US | To compare mortality for Caucasians and African-Americans in non-dialysis dependent CKD | 570808 | US veterans with non-dialysis dependent CKD | CKD stages 3-5 | African-Americans, Caucasians | historical cohort | Cox proportional hazards regression model | all-cause mortality | age, sex, marital and insurance status, geographic region, blood pressure, diabetes, cardiovascular disease, peripheral vascular disease, cerebrovascular disease, congestive heart failure, Charlson comorbidity index, laboratory variables ( eGFR, serum albumin, cholesterol, haemoglobin, white blood cell count and serum alkaline phosphatase) | African-Americans race was associated with significantly lower crude mortality (HR, 0.95; 95% CI, 0.94–0.97; p<0.001). The survival advantage was attenuated after adjustment for age (HR, 1.14; 95% CI, 1.12–1.16), sociodemographic characteristics, comorbidities and laboratory findings resulted in a significant reduction in mortality risk associated with African-Americans race (HR, 0.72; 95% CI, 0.70–0.73). The crude survival advantage of African-Americans patients was least present in patients with CKD stage 3a (eGFR, 45–59 ml/min/1.73m2) and increased linearly with CKD stages 3b through 5 |
| Mathur et al., 2018 | UK | To determine ethnic differences in the progression of chronic kidney disease (CKD) and risk of end-stage renal failure (ESRF) and death in adults with type 2 diabetes mellitus (T2DM), and to identify predictors of rapid renal decline. | 6274 | General practice-registered adults aged 25–85 years with established T2DM and CKD at baseline. | CKD stage 3 or below | Caucasian, South Asian, Afro-Caribbean | Prospective cohort study with nested case-control study | multilevel linear regression (rate of decline); logistic regression (odds of decline); Cox proportional hazards regression | Progression to ESRD, GFR slope | age, gender, Index of Multiple Deprivation score, systolic blood pressure (SBP), glycated haemoglobin (HbA1c), proteinuria, smoking status, BMI, diagnosed cardiovascular disease (ischaemic heart disease, heart failure, stroke, peripheral vascular disease) and any prescription of ACE inhibitors and angiotensin receptor blockers (ARBs). | Age-sex adjusted rate of eGFR decline was fastest in South Asian (coeff (95% CI): -0.77(-0.81,-0.74) than Caucasians (-0.64 (-0.68,-0.60) and Afro-Caribbeans (0.55(-0.61,-0.48). After adjusting for confounders, African-Caribbean and South Asians had higher odds of faster progression. Adjusted OR for African-Caribbean compared to Caucasian: 2.53 (95% CI: 1.63-3.92). The risk of ESRD was more than doubled in African-Americans compared to Caucasians in a fully adjusted analysis which also took account of the competing risk of death (HR 2.23, 95% CI 1.25 to 2.98). Adjusted risk for death, compared to Caucasians, was HR (95% CI): 0.71 (0.56 to 0.90) and 0.77 (0.57 to1.05) for South Asian and Afro-Caribbeans, respectively. |
| Mehrotra et al., 2008 | US | evaluate racial differences in mortality in early stages of CKD | 14611 | community dwelling adults (aged ≥20 years) in the Third National Health and Nutrition Examination Survey (NHANES III) | GFR <60 ml/min per 1.73 m^2^ | Caucasian, African-Americans, Mexican American | Prospective cohort study | Cox proportional hazards regression model | all-cause and cardiovascular related mortality | age, sex, socioeconomic status, CKD stage, cardiovascular risk factors | In the subgroup with CKD, adjusting for age and gender, African-Americans individuals had a significantly higher risk for all-cause mortality (HR(95% CI): 1.78 (1.14 to 2.78))(but not CVD mortality), and this risk was modified by age; specifically, African-Americans individuals who were younger than 65 years were 78% more likely to die than Caucasian individuals, whereas no significant differences in mortality were observed among individuals who were > 65 years of age. Adjusting for CVD risk factors and CKD stage did not affect results, but adjusting for SES factors resulted in non-significant effect of race on death (adjusted HR(95% CI): 1.38 (0.83 to 2.28)) |
| Menon et al., 2008 | US | Examine renal outcomes in non-diabetic participants with CKD stage 2-4 | 1666 | Nondiabetic participants from the Modification of Diet in Renal Disease (MDRD) study | CKD stage 2-4 | African-Americans, non-African-Americans | Retrospective cohort study | Competing risk models | Kidney failure, mortality | Subgroup analysis by GFR, proteinuria, kidney disease aetiology, sex | Rates for kidney failure and death (both before and subsequent to kidney failure) were similar for men and women, and for African-Americans versus non-African-Americans, and kidney failure was the more likely outcome in each of these groups. Rate (per 1000 person-years, 95% CI) for kidney failure was 85 (79-90) and 80 (65-96) for Caucasians and African-Americans, respectively. Rate (per 1000 person-years, 95% CI) for death was 23 (20-25) and 25 (18-32) for Caucasians and African-Americans, respectively. |
| Navaneethan et al., 2011 | US | Assess the associations between low 25 hydroxyvitamin D (25[OH]D) levels and mortality among non-dialysis dependent CKD | 12763 | Cleveland Clinic health care outpatients | CKD stages 3-4 | African-Americans, non-African Americans | retrospective cohort study | Logistic regression models; Cox proportional hazards model | mortality | age, sex, BMI, eGFR, diabetes, hypertension, hyperlipidaemia, malignancy, congestive heart failure, cerebrovascular disease, coronary artery disease, season of 25(OH)D testing, serum albumin and haemoglobin and year of entry into our registry | 25(OH) D <15 ng/ml was independently associated with all-cause mortality in non-dialysis dependent CKD. Race (OR: 1.81 (1.60-2.05 for African-Americans compared to Caucasians), diabetes, hypertension, cerebrovascular disease and season of vitamin D testing were not significantly associated with increased mortality |
| Newsome et al., 2006 | US | to assess, in a nationally representative sample of patients with cardiovascular disease, ethnic differences in survival among pre-dialysis patients with kidney disease | 57942 | patients hospitalised with acute myocardial infarction between 1994-1995 | CKD stages 1-4 | African-Americans, Caucasians | retrospective cohort study | Cox proportional hazards model | 3 year mortality | gender, age, smoking, status, comorbidities (history of hypertension, congestive heart failure, diabetes, and stroke), clinical presentation (Acute Physiology and Chronic Health Evaluation [APACHE] II score, cardiac arrest, and haematocrit), patient treatment characteristics (aspirin prescription, blocker prescription, angiotensin-converting enzyme inhibitor prescription upon discharge, and reperfusion during hospitalization), and hospital procedural capabilities | The African-Americans versus Caucasian hazard ratio for mortality among patients with a GFR from 15 to 30 ml/min per 1.73 m2 was 0.79 (95% confidence interval 0.61 to 0.97). Among patients with incident acute myocardial infarction, African-Americans patients with more severe kidney disease, when compared with their Caucasian counterparts, experience better survival |
| Norris et al., 2008 | US | Commentary on studies exploring ethnic differences in CKD progression and mortality |  | African-Americans and Caucasians in the general population | All stages | non-Hispanic Caucasian, African American, Asian, American Indian/Alaska Native, and Hispanic | Commentary | n/a | CKD progression to end-stage renal disease, cardiovascular and non-cardiovascular-related mortality |  | Biological, environmental, sociocultural, and healthcare system factors contribute to ethnic disparities in CKD outcomes |
| Parsa et al., 2013 | US | Examine the effects of variants in the gene encoding apolipoprotein L1 (APOL1) on the progression of chronic kidney disease in Caucasian and African-American people | 2955 | Patients from the African American Study of Kidney Disease and Hypertension (AASK) and Chronic Renal Insufficiency Cohort (CRIC) | eGFR: 20-70ml/min/1.73m^2^ | African-Americans, Caucasians | Prospective cohort study | Mixed-effects and Cox proportional-hazards model | Rate of eGFR decline, a composite of ESRD and 50% reduction in baseline eGFR, mortality | Age, sex, clinical site, and baseline eGFR, education level, treatment by a nephrologist, and use of either an ACE inhibitor or angiotensin-receptor blocker [as a proxy for treatment access, systolic blood pressure, body-mass index, glycated haemoglobin level, and smoking status, total 24-hour urinary protein excretion | African-American patients in the APOL1 high-risk group had a more rapid decline in eGFR and a higher risk of the composite renal outcome than did Caucasian patients and African-American patients in the low APOL1 risk group, among those with diabetes (-0.79 (-1.41 to -0.17)ml/min/1.73m^2^ )for African-Americans compared to Caucasians, p=0.01) and those without diabetes (-0.81 (-1.26 to -0.35) ml/min/1.73m2 for African-Americans compared to Caucasians). Death rates for African-American patients were similar to those for Caucasian patients. |
| Peralta et al., 2006 | US | Examine the associations between Hispanic ethnicity and risks for ESRD, cardiovascular events, and death in patients with CKD. | 39550 | Patients with stages 3 to 4 CKD from Kaiser Permanente of Northern California | CKD stages 3 to 4 | Hispanics, non-Hispanic Caucasians | Prospective cohort study | Cox regression models | ESRD, cardiovascular events, mortality | Age, gender, income, education, and preferred language, hypertension and medical history, diabetes and use of insulin, baseline eGFRc and time-updated proteinuria, time-varying medication used | After adjustment for sociodemographic characteristics, Hispanic ethnicity was associated with an increased risk for ESRD (hazard ratio [HR] 1.93; 95% confidence interval [CI] 1.72 to 2.17) when compared with non-Hispanic Caucasian patients, which was attenuated after controlling for diabetes and insulin use (HR 1.50; 95% CI 1.33 to 1.69). After further adjustment for potential confounders, Hispanic ethnicity remained independently associated with an increased risk for ESRD (HR 1.33; 95% CI 1.17 to 1.52) as well as a lower risk for cardiovascular events (HR 0.82; 95% CI 0.76 to 0.88) and death (HR 0.72; 95% CI 0.66 to 0.79). |
| Weiner et al., 2004 | US | assess whether CKD is a risk factors for cardiovascular disease | 22,634 | participants aged 45 to 64 years from Jackson, Mississippi; Forsyth County, North Carolina; the north-western suburbs of Minneapolis, Minnesota; and Washington County, Maryland | eGFR between 15 and 60 ml/min per 1.73 m^2^ | African-American ,Caucasian | Retrospective cohort study | Cox proportional hazards model | cardiac event, stroke, composite score of death, nonfatal myocardial infarction and stroke, mortality | age, gender, education status, alcohol use, smoking status, BMI, systolic BP, total cholesterol, HDL, cholesterol, diabetes, and history of hypertension, LVH | greater risk of the composite end point of death, nonfatal myocardial infarction, and stroke among African Americans than among Caucasians, due in part to a greater severity of hypertension and/or diabetes in the former. All-cause mortality was higher in African Americans (HR, 1.83; 95% CI, 1.33–2.52) versus Caucasians (HR, 1.31; 95% CI, 1.16–1.49). |
| Wetmore et al., 2011 | US | to examine racial differences in the prevalence or prognostic importance of renal insufficiency in acute myocardial infarction (AMI) | 1847 | patients (aged 18+ years) with acute myocardial infarction and enrolled in the multicentre Prospective Registry Evaluating Myocardial Infarction Event and Recovery (PREMIER) study | eGFR: 30-59.9 ml/min per 1.73 m^2^, <30 ml/min per 1.73 m^2^, | African American, Caucasian | prospective cohort study | multivariable proportional hazards regression | 3.5-year all-cause mortality | eGFR, demographic (age, sex), socioeconomic factor (education), risk behaviour (smoking status), comorbidity (prior coronary artery disease, prior cerebrovascular accident, congestive heart failure, hypertension, hypercholesterolemia, diabetes mellitus, chronic lung disease, cancer, and body mass index), AMI type (ST-elevation AMI versus not), acute non-cardiac condition warranting admission (23), left ventricular systolic function ( 40% versus not), acute heart rate, and laboratory variables (initial haematocrit and glucose). | Among those with eGFR <60ml/min/1.73m2, risk of mortality was higher in African-Americans than Caucasians though this was not significantly different (HR 1.20, 95% CI 0.90 to 1.62). . |
| Yang et al., 2014 | US | Assess association of risk factors with CKD progression indicated by ESRD, eGFR decline or death | 3939 | Adults aged 21 to 74 and with CKD (Chronic Renal Insufficiency Cohort (CRIC) study) | Mild to moderate CKD | Caucasian, African-American, other | Prospective cohort study | [Multivariable Cox proportional hazards models](https://www.ncbi.nlm.nih.gov/pmc/articles/PMC3946885/#R14) | eGFR decline, ESRD, mortality | Age, race, sex, diabetes, baseline estimated glomerular filtration rate (eGFR), proteinuria, ankle brachial index, uric acid, history of CVD, BMI, hypertension, haemoglobin, education and smoking status. | Older age, diabetes, lower eGFR, and high proteinuria levels were associated with increased risk of death, female sex associated with lower risk of death and no significant association between race and risk of death (HR (95% CI): 1.09 (0.89,1.33) for African-Americans compared to Caucasians). Older age, female sex were associated with lower rates of ESRD. The HRs for ESRD among African-American and those of other races/ethnicity compared to Caucasians were 1.55 (95% CI, 1.29–1.86) and 1.52 (95% CI, 1.17–1.98), respectively. The HRs for eGFR halving among African-American and those of other races/ethnicity compared to Caucasians were 1.43 (95% CI, 1.22–1.68) and 1.44 (95% CI, 1.14–1.82), respectively |
